# Meta-Analyses Support a Taxonomic Model for Representations of Different Categories of Audio-Visual Interaction Events in the Human Brain

**DOI:** 10.1093/texcom/tgab002

**Published:** 2021-01-18

**Authors:** Matt Csonka, Nadia Mardmomen, Paula J Webster, Julie A Brefczynski-Lewis, Chris Frum, James W Lewis

**Affiliations:** Department of Neuroscience, Rockefeller Neuroscience Institute, West Virginia University, Morgantown, WV 26506, USA; Department of Neuroscience, Rockefeller Neuroscience Institute, West Virginia University, Morgantown, WV 26506, USA; Department of Neuroscience, Rockefeller Neuroscience Institute, West Virginia University, Morgantown, WV 26506, USA; Department of Neuroscience, Rockefeller Neuroscience Institute, West Virginia University, Morgantown, WV 26506, USA; Department of Neuroscience, Rockefeller Neuroscience Institute, West Virginia University, Morgantown, WV 26506, USA; Department of Neuroscience, Rockefeller Neuroscience Institute, West Virginia University, Morgantown, WV 26506, USA

**Keywords:** categorical perception, embodied cognition, multisensory integration, neuroimaging, sensory-semantic categories

## Abstract

Our ability to perceive meaningful action events involving objects, people, and other animate agents is characterized in part by an interplay of visual and auditory sensory processing and their cross-modal interactions. However, this multisensory ability can be altered or dysfunctional in some hearing and sighted individuals, and in some clinical populations. The present meta-analysis sought to test current hypotheses regarding neurobiological architectures that may mediate audio-visual multisensory processing. Reported coordinates from 82 neuroimaging studies (137 experiments) that revealed some form of audio-visual interaction in discrete brain regions were compiled, converted to a common coordinate space, and then organized along specific categorical dimensions to generate activation likelihood estimate (ALE) brain maps and various contrasts of those derived maps. The results revealed brain regions (cortical “hubs”) preferentially involved in multisensory processing along different stimulus category dimensions, including 1) living versus nonliving audio-visual events, 2) audio-visual events involving vocalizations versus actions by living sources, 3) emotionally valent events, and 4) dynamic-visual versus static-visual audio-visual stimuli. These meta-analysis results are discussed in the context of neurocomputational theories of semantic knowledge representations and perception, and the brain volumes of interest are available for download to facilitate data interpretation for future neuroimaging studies.

## Introduction

The perception of different categories of visual (unisensory) object and action forms are known to differentially engage distinct brain regions or networks in neurotypical individuals, such as when observing or identifying faces, body parts, living things, houses, fruits and vegetables, and outdoor scenes, among other proposed categories (Martin et al. 1996; Tranel et al. 1997; Caramazza and Mahon 2003; Martin 2007). Distinct semantic categories of real world sound-producing (unisensory) events are also known or thought to recruit different brain networks, such as nonliving environmental and mechanical sounds (Lewis et al. 2012), nonvocal action events produced by nonhuman animal sources (Engel et al. 2009; Lewis, Talkington, et al. 2011), as well as the more commonly studied categories of living things (especially human conspecifics) and vocalizations (notably speech) (Dick et al. 2007; Saygin et al. 2010; Goll et al. 2011; Trumpp et al. 2013; Brefczynski-Lewis and Lewis 2017). Extending beyond unisensory category-specific percepts, the neurobiological representations of multisensory events are thought to develop based on complex combinations of sensory and sensory-motor information, with some dependence on differences with individual observers’ experiences throughout life, such as with handedness (Lewis et al. 2006). One may have varying experiences with, for instance, observing and hearing a construction worker hammering a nail, or feeling a warm purring gray boots breed cat on a sofa. Additionally, while watching television, or a smart phone device, one can readily accept the illusion that the synchronized audio (speakers) and video movements (the screen) are emanating from a single animate or object source, leading to stable, unified multisensory percepts. Psychological literature indicates that perception of multisensory events can manifest as well-defined category-specific objects and action representations that build on past experiences (Rosch 1973; Vygotsky 1978; McClelland and Rogers 2003; Miller et al. 2003; Martin 2007). However, the rules that may guide the organization of cortical network representations that mediate multisensory perception of real-world events, and whether any taxonomic organizations for such representations exist at a categorical level, remain unclear.

The ability to organize information to attain a sense of global coherence, meaningfulness, and possible intention behind every-day observable events may fail to fully or properly develop, as for some individuals with autism spectrum disorder (ASD) (Jolliffe and Baron-Cohen 2000; Happe and Frith 2006; Kouijzer et al. 2009; Powers et al. 2009; Marco et al. 2011; Pfeiffer et al. 2011, 2018; Ramot et al. 2017; Webster et al. 2020) and possibly for some individuals with various forms of schizophrenia (Straube et al. 2014; Cecere et al. 2016; Roa Romero et al. 2016; Vanes et al. 2016). Additionally, brain damage, such as with stroke, has been reported to lead to deficits in multisensory processing (Van der Stoep et al. 2019). Thus, further understanding the organization of the multisensory brain has been becoming a topic of increasing clinical relevance.

At some processing stages or levels, the central nervous system is presumably “prewired” to readily develop an organized architecture that can rapidly and efficiently extract meaningfulness from multisensory events. This includes audio-visual event encoding and decoding that enables a deeper understanding of one’s environment, thereby conferring a survival advantage through improvements in perceived threat detection and in social communication (Hewes 1973; Donald 1991; Rilling 2008; Robertson and Baron-Cohen 2017). An understanding of multisensory neuronal processing mechanisms, however, may in many ways be better understood through models of semantic knowledge processing rather than models of bottom-up signal processing, which is prevalent in unisensory fields of literature. One set of theories behind semantic knowledge representation includes distributed-only views, wherein auditory, visual, tactile, and other sensory-semantic systems are distributed neuroanatomically with additional task-dependent representations or convergence-zones in cortex that link knowledge (Damasio 1989a; Languis and Miller 1992; Damasio et al. 1996; Tranel et al. 1997; Ghazanfar and Schroeder 2006; Martin 2007). A distributed-plus-hub view further posits the existence of additional task-independent representations (or “hubs”) that support the interactive activation of representations in all modalities, and for all semantic categories (Patterson et al. 2007).

More recent neurocomputational theories of semantic knowledge learning entails a sensory-motor framework wherein action perception circuits (APCs) are formed through sensory experiences, which manifest as specific distributions across cortical areas (Pulvermuller 2013, 2018; Tomasello et al. 2017). In this construct, combinatorial knowledge is thought to become organized by connections and dynamics between APCs, and cognitive processes can be modeled forthright. Such models have helped to account for the common observation of cortical hubs or “connector hubs” for semantic processing (Damasio 1989b; Sporns et al. 2007; van den Heuvel and Sporns 2013), which may represent multimodal, supramodal, or amodal mechanisms for representing knowledge. From this connector hub theoretical perspective, it remains unclear whether or how different semantic categories of multisensory perceptual knowledge might be organized, potentially including semantic hubs that link, for instance, auditory and visual unisensory systems at a category level.

Here, we addressed the issue of global neuronal organizations that mediate different aspects of audio-visual categorical perception by using activation likelihood estimate (ALE) meta-analyses of a diverse range of published studies to date that reported audio-visual interactions of some sort in the human brain. We defined the term “interaction” to include measures of neuronal sensitivity to temporal and/or spatial correspondence, response facilitation or suppression, inverse effectiveness, an explicit comparison of information from different modalities that pertained to a distinct object, and cross-modal priming (Stein and Meredith 1990; Stein and Wallace 1996; Calvert and Lewis 2004). These interaction effects were assessed in neurotypical adults (predominantly, if not exclusively, right-handed) using hemodynamic blood flow measures (functional magnetic resonance imaging [fMRI], or positron emission tomography [PET]) or magnetoencephalography (MEG) methodologies as whole brain neuroimaging techniques.

The resulting descriptive compilations and analytic contrasts of audio-visual interaction sites across different categories of audio-visual stimuli were intended to meet three main goals: The first goal was to reveal a global set of brain regions (cortical and noncortical) with significantly high probability of cross-sensory interaction processing regardless of variations in methods, stimuli, tasks, and experimental paradigms. The second goal was to validate and refine earlier multisensory processing concepts borne out of image-based meta-analyses of audio-visual interaction sites (Lewis 2010) that used a subset of the paradigms included in the present study, but here taking advantage of coordinate-based meta-analyses and more rigorous statistical approaches now that additional audio-visual interaction studies have subsequently been published.

**Figure 1:**
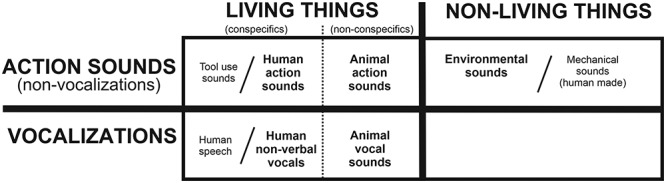
A taxonomic category model of the neurobiological organization of the human brain for processing and recognizing different acoustic–semantic categories of natural sounds (from Brefczynski-Lewis and Lewis 2017). Bold text in the boxed regions depict rudimentary sound categories, including living versus nonliving things and vocalizations versus nonvocal action sounds, which are categories being tested in the present audio-visual meta-analyses. Other subcategories are also indicated, including human speech, tool use sounds, and human-made machinery sounds. Vocal and instrumental music sounds/events are regarded as higher forms of communication, which rely on other networks and are thus outside the scope of the present study. Refer to text for other details.

The third goal, as a special focus, was to test recent hypotheses regarding putative brain architectures mediating multisensory categorical perception that were derived from unisensory auditory object perception literature (Fig. 1), which encompassed theories to explain how real-world natural sounds are processed to be perceived as meaningful events to the observer (Brefczynski-Lewis and Lewis 2017). This hearing perception model entailed four proposed tenets that may shape brain organizations for processing real-world natural sounds, helping to explain “why” certain category-preferential representations appear in the human brain (and perhaps more generally in the brains of all mammals with hearing ability). These tenets for hearing perception included: 1) parallel hierarchical pathways process increasing information content, 2) metamodal operators guide sensory and multisensory processing network organizations, 3) natural sounds are embodied when possible, and 4) categorical perception emerges in neurotypical listeners.

After compiling the numerous multisensory human neuroimaging studies that employed different types of audio-visual stimuli, tasks, and imaging modalities, we sought to test three hypotheses relating to the above mentioned tenets and neurobiological model. The first two hypotheses effectively tested for support of the major taxonomic boundaries depicted in Figure 1: The first hypothesis being 1) that there will be a double-dissociation of brain systems for processing living versus nonliving audio-visual events, and the second hypothesis 2) that there will be a double-dissociation for processing vocalizations versus action audio-visual events produced by living things.

In the course of compiling neuroimaging literature, there was a clear divide between studies using static visual images (iconic representations) versus video with dynamic motion stimuli that corresponded with aspects of the auditory stimuli. The production of sound necessarily implies dynamic motion of some sort, which in many of the studies’ experimental paradigms also correlated with viewable object or agent movements. Thus, temporal and/or spatial intermodal invariant cues that physically correlate visual motion (“dynamic-visual”) with changes in acoustic energy are typically uniquely present in experimental paradigms using video (Stein and Meredith 1993; Lewkowicz 2000; Bulkin and Groh 2006). Conversely, static or iconic visual stimuli (“static-visual”) must be learned to be associated and semantically congruent with characteristic sounds, and with varying degrees of arbitrariness. Thus, a third hypothesis emerged 3) that the processing of audio-visual stimuli that entailed dynamic-visual motion stimuli versus static-visual stimuli will also reveal a double-dissociation of cortical processing pathways in the multisensory brain. The identification and characterization of any of these hypothesized neurobiological processing categories at a meta-analysis level would newly inform neurocognitive theories, specifying regions or network hubs where certain types of information may merge or in some way interact across sensory systems at a semantic category level. Thus, the resulting ALE maps are expected to facilitate the generation of new hypotheses regarding multisensory interaction and integration mechanisms in neurotypical individuals. They should also contribute to providing a foundation for ultimately understanding “why” multisensory processing networks develop the way they typically do, and why they may develop aberrantly, or fail to recover after brain injury, in certain clinical populations.

## Materials and Methods

**Figure 2:**
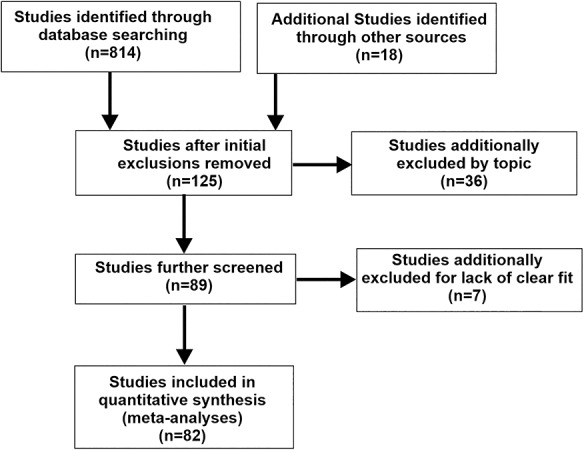
PRISMA table illustrating the flow of information through the different phases of the meta-analysis review.

This work was performed in accordance with the PRISMA statement for reporting systematic reviews and meta-analyses of studies that evaluate health care interventions (Moher et al. 2009). Depicted in the PRISMA flow-chart (Fig. 2), original research studies were identified by PubMed and Google Scholar literature searches with keyword combinations “auditory + visual,” “audiovisual,” “multisensory,” and “fMRI” or “PET” or “MEG,” supplemented through studies identified through knowledge of the field published between 1999 through early 2020. Studies involving drug manipulations, patient populations, children, or nonhuman primates were excluded unless there was a neurotypical adult control group with separately reported outcomes. Of the included studies, reported coordinates for some paradigms had to be estimated from figures. Additionally, some studies did not use whole-brain imaging, but rather incorporated imaging to 50–60 mm slabs of axial brain slices so as to focus, for instance, on the thalamus or basal ganglia. These studies were included despite their being a potential violation of assumptions made by ALE analyses (see below) because the emphasis of the present study was to reveal proof of concept regarding differential audio-visual processing at a semantic category level. This yielded inclusion of 82 published fMRI, PET and MEG studies including audio-visual interaction(s) of some form (Table 1). The compiled coordinates, after converting to afni-TLRC coordinate system, derived from these studies are included in Appendix A, and correspond directly to Table 1.

**Table 1:** List of all studies used in the subsequent subsets of audio-visual interaction site meta-analyses

Study #	Experiment #				# Subjects	Multiple experiments	Left hem foci	Right hem foci	Number of foci		Congruent versus Incong	Living versus Nonliving	Emotional stimuli/task	Vocalizations versus Not	Dynamic (video) versus static
82	137	First author	Year	Experimental code and abbreviated task	1285		376	338	714	Brief description of experimental paradigm	2B	2C	2C	2D	2E
1	1	Adams	2002	Expt 1 Table 3 A + V (aud coords only)	12		5	1	6	A and V commonly showing subordinate > basic object name verification (words with pictures or environmental sounds)	1	2		2	2
2	2	Alink	2008	Table 1c spheres move to drum sounds	10		4	6	10	Visual spheres and drum sounds moving: crossmodal dynamic capture versus conflicting motion	1	2		2	1
3	3	Balk	2010	Figure 2 asynchronous versus simultaneous	14		2	1	3	Natural asynchronous versus simultaneous AV speech synchrony (included both contrasts as interaction effects)	1			1	1
4	4	Baumann	2007	Table 1B coherent V + A versus A	12		2	1	3	Visual dots 16% coherent motion and in-phase acoustic noise > stationary acoustic sound	1	2	2	2	1
	5	Baumann	2007	Table 2B		pooled	15	12	27	Moving acoustic noise and visual dots 16% in-phase coherent > random dot motion	1	2	2	2	1
5	6	Baumgaertner	2007	Table 3 Action > nonact sentence+video	19		3	0	3	Conjunction spoken sentences (actions > nonactions) AND videos (actions > nonactions)	1	1		1	1
6	7	Beauchamp	2004a	Figure 3*J* and *K*, Table 1 first 2 foci only	26		2	0	2	See photographs of tools, animals and hear corresponding sounds versus scrambled images and synthesized rippled sounds	1	1	2	2	2
7	8	Beauchamp	2004b	Expt 1 coordinates	8		1	1	2	High resolution version of 2004a study: AV tool videos versus unimodal (AV > A,V)	1	1	2	2	1
8	9	Belardinelli	2004	Table 1 AV semantic congruence	13		6	6	12	Colored images of tools, animals, humans and semantically congruent versus incongruent sounds	1	1		2	2
	10	Belardinelli	2004	Table 2 AV semantic incongruent		pooled	2	3	5	Colored images of tools, animals, humans and semantically incongruent versus congruent sounds	2	0	0	0	0
9	11	Biau	2016	Table 1A Interaction; speech synchronous	17		8	0	8	Hand gesture beats versus cartoon disk and speech interaction: synchronous versus asynchronous	1	1		1	1
10	12	Bischoff	2007	Table 2A only *P* < 0.05 included	19		2	1	3	Ventriloquism effect: gray disks and tones, synchronous (*P* < 0.05 corrected)	1			2	
11	13	Blank	2013	Figure 2	19		1	0	1	Visual-speech recognition correlated with recognition performance	1	1		1	1
12	14	Bonath	2013	pg 116 congruent thalamus	18		1	0	1	Small checkerboards and tones: spatially congruent versus incongruent (thalamus)	1	2	2	2	
	15	Bonath	2013	pg 116 incongruent		pooled	1	1	2	Small checkerboards and tones: spatially incongruent versus congruent (thalamus)	2	0	0	0	0
13	16	Bonath	2014	Table 1A illusory versus not	20		1	5	6	Small checkerboards and tones: temporal > spatial congruence	1	2	2	2	
	17	Bonath	2014	Table 1B synchronous > no illusion		pooled	3	0	3	Small checkerboards and tones: spatial > temporal congruence	1	2	2	2	
14	18	Bushara	2001	Table 1A (Fig. 2) AV-Control	12		1	3	4	Tones (100 ms) and colored circles synchrony: detect Auditory then Visual presentation versus Control	1	2		2	
	19	Bushara	2001	Table 1B (VA-C) five coords		pooled	2	3	5	Tones (100 ms) and colored circles synchrony: detect Visual then Auditory presentation versus Control	1	2		2	
	20	Bushara	2001	Table 2A interact w/Rt Insula		pooled	2	4	6	Tones and colored circles: correlated functional connections with (and including) the right insula	1	2		2	
15	21	Bushara	2003	Table 2A collide > pass, strong A-V interact	7		5	3	8	Tone and two visual bars moving: tone synchrony induce perception they collide (AV interaction) versus pass by	1	2		2	1
16	22	Callan	2014	Table 5 AV-Audio (AV10-A10)-(AV6-A6)	16		4	4	8	Multisensory enhancement to visual speech in noise correlated with behavioral results	1	1		1	1
	23	Callan	2014	Table 6 AV—Visual only		pooled	1	1	2	Multisensory enhancement to visual speech audio-visual versus visual only	1	1		1	1
17	24	Calvert	1999	Table 1 (Fig. 1)	5		3	4	7	View image of lower face and hear numbers 1 through 10 versus unimodal conditions (AV > Photos, Auditory)	1	1	2	1	2
18	25	Calvert	2000	Figure 2 superadditive+subadditive AVspeech	10		1	0	1	Speech and lower face: supra-additive plus subadditive effects (AV-congruent > A,V > AV-incongruent)	1	1	2	1	1
	26	Calvert	2000	Table 1 supradditive AVspeech		pooled	4	5	9	Speech and lower face: supra-additive AV enhancement	1	1	2	1	1
	27	Calvert	2000	Table 2 incongruent subadditive AVspeech		pooled	3	3	6	Speech and lower face: subadditive AV response to incongruent AV inputs	2	0	0	0	0
19	28	Calvert	2001	Table 2 superadditive and response depression	10		4	11	15	B/W visual checkerboard reversing and white noise bursts: Synchronous versus not; supradditive and response depression	1	2		2	
	29	Calvert	2001	Table 3A superadditive only		pooled	6	4	10	B/W visual checkerboard reversing and white noise bursts: Synchronous versus not; supradditive only	1	2		2	
	30	Calvert	2001	Table 3B response depression only		pooled	3	4	7	B/W visual checkerboard reversing and white noise bursts: Synchronous versus not; response depression only	1	2		2	
20	31	Calvert	2003	Table 2A (Fig. 3 blue)	8		13	8	21	Speech and lower face: Moving dynamic speech (phonemes) versus stilled speech frames	1	1	2	1	1
21	32	DeHaas	2013	Table 1A AVcong—Visual	15		3	3	6	Video clips of natural scenes (animals, humans): AV congruent versus Visual	1	1			1
	33	DeHaas	2013	Table 1B V-AV incongruent		pooled	2	0	2	Video clips of natural scenes (animals, humans): Visual versus AV incongruent	2	0	0	0	0
22	34	Erickson	2014	Table 1A Congruent AV speech	10		2	2	4	McGurk effect (phonemes): congruent AV speech: AV > A and AV > V	1	1	2	1	1
	35	Erickson	2014	Table 1B McGurk speech		pooled	2	0	2	McGurk speech effect (phonemes)	1	1	2	1	1
23	36	Ethofer	2013	Table 1C emotion	23		1	2	3	Audiovisual emotional face-voice integration	1	1	1	1	1
24	37	Gonzalo	2000	Table 1 AV > AVincon music and Chinese ideograms	14		1	1	2	Learn novel Kanji characters and musical chords, activity increases over time for consistent AV pairings	1			2	2
	38	Gonzalo	2000	Table 2 inconsistent AV		pooled	4	4	8	Learn novel Kanji characters and musical chords, activity increases over time to inconsistent pairings	2	0	0	0	0
	39	Gonzalo	2000	Table 3 AV consistent versus Aud		pooled	1	1	2	Learn novel Kanji characters and musical chords, learn consistent (vs. inconsistent) pairings versus auditory only	1			2	2
25	40	Green	2009	Table 1 incongruent > congruent gesture-speech	16		4	5	9	Incongruent versus congruent gesture-speech	2	1		1	1
	41	Green	2009	Table 4A Congruent gesture-speech > gesture or speech		pooled	1	0	1	Congruent gesture-speech versus gesture with unfamiliar speech and with familiar speech	1	1		1	1
26	42	Hagan	2013	Table 1 AV emotion, novel over time	18		5	3	8	Affective audio-visual speech: congruent AV emotion versus A, V; unique ROIs over time (MEG)	1		1	1	1
	43	Hagan	2013	Table 2 AV emotion incongruent		pooled	1	5	6	Affective audio-visual speech: incongruent AV emotion versus A, V; unique ROIs over time (MEG)	2	0	0	0	0
27	44	Hasegawa	2004	Table 1A (well trained piano) AV induced by V-only	26		12	6	18	Piano playing: well trained pianists, mapping hand movements to sequences of sound	1	1		2	1
28	45	Hashimoto	2004	Table 1G (Fig. 4B, red) Learning Hangul letters to sounds	12		2	1	3	Unfamiliar Hangul letters and nonsense words, learn speech versus tone/noise pairings	1		2	1	2
29	46	He	2015	Table 3C AV speech foreign (left MTG focus)	20		1	0	1	Intrinsically meaningful gestures with German speech: Gesture-German > Gesture-Russian, German speech only	1	1		1	1
30	47	He	2018	Table 2 gestures and speech integration	20		1	0	1	Gesture-speech integration: Bimodal speech-gesture versus unimodal gesture with foreign speech and versus unimodal speech	1	1	0	1	1
31	48	Hein	2007	Figure 2A AV incongruent	18		0	2	2	Familiar animal images and incorrect (incongruent) vocalizations (dog: meow) versus correct pairs	2	0	0	0	0
	49	Hein	2007	Figure 2B AV-artificial/nonliving		pooled	0	1	1	B/W images of artificial objects (“fribbles”) and animal vocalizations versus unimodal A, V	1				2
	50	Hein	2007	Figure 2C pSTS, pSTG, mSTG AV-cong		pooled	0	3	3	Familiar animal images and correct vocalizations (dog: woof-woof)	1	1		1	2
	51	Hein	2007	Figure 3A incongruent		pooled	4	0	4	AV familiar incongruent versus unfamiliar artificial (red foci 1, 5, 6, 9)	2	0	0	0	0
	52	Hein	2007	Figure 3B Foci 2, 3, 4 (blue) artificial/nonliving		pooled	3	0	3	Visual “Fribbles” and backward/underwater distorted animal sounds, learn pairings (blue foci 2,3,4)	1			1	2
	53	Hein	2007	Figure 3C congruent living (green)		pooled	3	0	3	Familiar congruent living versus artificial AV object features and animal sounds (green foci 7, 8, 10)	1			1	2
32	54	Hocking	2008	pg 2444 verbal	18		2	0	2	(pSTS mask) Color photos, written names, auditory names, environmental sounds conceptually matched “amodal”	1	1			2
	55	Hocking	2008	Table 3 incongruent simultaneous matching		pooled	8	10	18	Incongruent sequential AV pairs (e.g., see drum, hear bagpipes) versus congruent pairs	2	0	0	0	0
33	56	Hove	2013	pg 316 AV interaction putamen	14		0	1	1	Interaction between (beep > flash) versus (siren > moving bar); left putamen focus	1	2			
34	57	James	2003	Figure 2	12		0	1	1	Activation by visual objects (“Greebles”) associated with auditory features (e.g., buzzes, screeches); (STG)	1				2
35	58	James	2011	Table 1*A* bimodal (vs. scrambled)	12		4	2	6	Video of human manual actions (e.g., sawing): Auditory and Visual intact versus scrambled, AV event selectivity	1	1	2	2	1
36	59	Jessen	2015	Table 1*A* emotion > neutral AV enhanced	17		1	1	2	Emotional multisensory whole body and voice expressions: AV emotion (anger and fear) > neutral expressions	1	1	1	1	1
	60	Jessen	2015	Table 1*D* fear > neutral AV enhanced		pooled	2	1	3	Emotional multisensory whole body and voice expressions: AV fear > neutral expressions	1	1	1	1	1
37	61	Jola	2013	Table 1*C* AVcondition dance	12		3	3	6	Viewing unfamiliar dance performance (tells a story by gesture) with versus without music: using intersubject correlation	1	1	1	2	1
38	62	Kim	2015	Table 2*A* AV > C speech semantic match	15		2	0	2	Moving audio-visual speech perception versus white noise and unopened mouth movements	1	1		1	1
39	63	Kircher	2009	Figure 3*B* gesture related activation increase	14		3	1	4	Bimodal gesture-speech versus gesture and versus speech	1	1		1	1
40	64	Kreifelts	2007	Table 1 voice-face emotion	24		1	2	3	Facial expression and intonated spoken words, judge emotion expressed (AV > A,V; *P* < 0.05 only)	1	1	1	1	1
	65	Kreifelts	2007	Table 5 AV increase effective connectivity		pooled	2	4	6	Increased effectiveness connectivity with pSTS and thalamus during AV integration of nonverbal emotional information	1	1	1	1	1
41	66	Lewis	2000	Table 1	7		2	3	5	Compare speed of tone sweeps to visual dot coherent motion: Bimodal versus unimodal	1	2		2	1
42	67	Matchin	2014	Table 1 AV > Aud only (McGurk)	20		2	7	9	McGurk audio-visual speech: AV > A only	1	1	2	1	1
	68	Matchin	2014	Table 2 AV > Video only		pooled	9	6	15	McGurk audio-visual speech: AV > V only	1	1	2	1	1
	69	Matchin	2014	Table 3 MM > AV McGurk		pooled	7	4	11	McGurk Mismatch > AV speech integration	2	0	0	0	0
43	70	McNamara	2008	Table (BA44 and IPL)	12		2	2	4	Videos of meaningless hand gestures and synthetic tone sounds: Increases in functional connectivity with learning	1			2	1
44	71	Meyer	2007	Table 3 paired A + V versus null	16		3	3	6	Paired screen red flashes with phone ring: paired V (conditioned stimulus) and A (unconditioned) versus null events	1	2		2	
	72	Meyer	2007	Table 4 CS+, learned AV association with V-only		pooled	4	6	10	Paired screen flashes with phone ring: View flashes after postconditioned versus null events	1	2		2	
45	73	Muller	2012	Table S1 effective connectivity changes	27		4	3	7	Emotional facial expression (groaning, laughing) AV integration and gating of information	1	1	1	1	2
46	74	Murase	2008	Figure 4 discordant > concordant AVinteraction	28		1	0	1	Audiovisual speech (syllables) showing activity to discordant versus concordant stimuli: left mid-STS	2	1	2	1	1
47	75	Naghavi	2007	Figure 1*C*	23		0	3	3	B/W pictures (animals, tools, instruments, vehicles) and their sounds: Congruent versus Incongruent	1	3		3	2
48	76	Naghavi	2011	Figure 2*A* cong = incon	30		1	0	1	B/W drawings of objects (living and non) and natural sounds (barking, piano): congruent = incongruent encoding	0				2
	77	Naghavi	2011	Figure 2*B* congruent > incongruent		pooled	0	1	1	B/W drawings of objects (living and non) and natural sounds (barking, piano): congruent > incongruent encoding	1				2
	78	Naghavi	2011	Figure 2*C* incongruent > congruent		pooled	1	1	2	B/W drawings of objects (living and non) and natural sounds (barking, piano): incongruent > congruent encoding	2	0	0	0	0
49	79	Nath	2012	pg 784	14		1	0	1	McGurk effect (phonemes): congruent AV speech correlated with behavioral percept	1	1	2	1	1
50	80	Naumer	2008	Figure 2 Table 1*A* max contrast	18		8	6	14	Images of “Fribbles” and learned artificial sounds (underwater animal vocals): post training versus max contrast	1			1	2
	81	Naumer	2008	Figure 3 Table 1*B* pre–post		pooled	5	6	11	Images of “Fribbles” and learned corresponding artificial sounds: Post- versus Pre-training session	1			1	2
	82	Naumer	2008	Figure 4 Table 2		pooled	1	1	2	Learn of “Freebles” and distorted sounds as incongruent > congruent pairs	2	0	0	0	0
51	83	Naumer	2011	Figure 3*C*	10		1	0	1	Photographs of objects (living and non) and related natural sounds	1				2
52	84	Noppeny	2008	Table 2 AV incongruent > congruent	17		5	2	7	Speech sound recognition through AV priming, environmental sounds and spoken words: Incongruent > congruent	2	0	0	0	0
	85	Noppeny	2008	Table 3 AV congruent sounds/words		pooled	4	0	4	Speech sound recognition through AV priming, environmental sounds and spoken words: Congruent > incongruent	1				2
53	86	Ogawa	2013a	Table 1 (pg 162 data)	13		1	0	1	AV congruency of pure tone and white dots moving on screen (area left V3A)	1	2		2	
54	87	Ogawa	2013b	Table 1 3D > 2D and surround > monaural effects	16		3	4	7	Cinematic 3D > 2D video and surround sound > monaural while watching a movie (“The Three Musketeers”)	1	1		0	1
55	88	Okada	2013	Table 1 AV > A	20		5	4	9	Video of AV > A speech only	1	1		1	1
56	89	Olson	2002	Table 1*A* synchronized AV > static Vis-only	10		7	4	11	Whole face video and heard words: Synchronized AV versus static V	1	1		1	1
	90	Olson	2002	Table 1*C* synchronized AV > desynchronized AV speech		pooled	2	0	2	Whole face video and heard words: Synchronized versus desynchronized	1	1		1	1
57	91	Plank	2012	pg 803 AV congruent effect	15		0	1	1	AV spatially congruent > semantically matching images of natural objects and associated sounds (right STG)	1	3		3	2
	92	Plank	2012	Table 2*A* spatially congruent-baseline		pooled	5	5	10	Images of natural objects and associated sounds, spatially congruent versus baseline	1	3		3	2
58	93	Raij	2000	Table 1*B* letters and speech sounds	9		2	3	5	Integration of visual letters and corresponding auditory phonetic expressions (MEG study) AV versus (A + V)	1		2	1	2
59	94	Regenbogen	2017	Table 2*A* degraded > clear Multisensory versus unimodal input	29		5	6	11	Degraded > clear AV versus both visual and auditory unimodal visual real-world object-in-action recognition	1	0		2	1
60	95	Robins	2008	Table 2 (Fig. 2) AV integration (AV > A and AV > V)	10		2	1	3	Face speaking sentences: angry, fearful, happy, neutral (AV > A,V)	1			1	1
	96	Robins	2008	Table 4A (Fig. 5) AV integration and emotion		pooled	1	4	5	AV faces and spoken sentences expressing fear or neutral valence: AV integration (AV > A,V conditions)	1	1		1	1
	97	Robins	2008	Table 4*B* emotion effects		pooled	2	0	2	AV faces and spoken sentences expressing fear or neutral valence: Emotional AV-fear > AV-neutral	1	1	1	1	1
	98	Robins	2008	Table 4*C* (Fig. 5) fearful AV integration		pooled	1	5	6	AV faces and spoken sentences expressing fear or neutral valence: Fearful-only AV integration	1	1	1	1	1
	99	Robins	2008	Table 4*D* AV-only emotion		pooled	1	3	4	AV faces and spoken sentences expressing fear or neutral valence: AV-only emotion	1	1	1	1	1
61	100	Scheef	2009	Table 1 cartoon jump + boing	16		1	2	3	Video of cartoon person jumping and “sonification” of a tone, learn correlated pairings: AV-V and AV-A conjunction	1	1	2	2	1
62	101	Schmid	2011	Table 2*E* A effect V (Living and nonliving, pictures)	12		3	4	7	Environmental sounds and matching pictures: reduced activity by A	1	3		3	2
	102	Schmid	2011	Table 2*F* V competition effect A (reduced activity by a visual object)		pooled	2	2	4	Environmental sounds and matching pictures: reduced activity by V	1	3		3	2
	103	Schmid	2011	Table 2*G* AV crossmodal interaction × auditory attention		pooled	2	3	5	Environmental sounds and matching pictures: cross-modal interaction and auditory attention	1	3		3	2
63	104	Sekiyama	2003	Table 3 (fMRI nAV-AV)	8		1	0	1	AV speech, McGurk effect with phonemes (ba, da, ga) and noise modulation: noise-AV > AV (fMRI)	1	1	2	1	1
	105	Sekiyama	2003	Table 4 (PET nAV-AV)		pooled	1	3	4	AV speech, McGurk effect with phonemes (ba, da, ga) and noise modulation: noise-AV > AV (PET)	1	1	2	1	1
64	106	Sestieri	2006	Table 1 (Fig. 3), AV location match versus semantic	10		2	5	7	B/W images (animal, weapons) and environmental sounds: Match location > recognition	1	1		2	2
	107	Sestieri	2006	Table 2 AV semantic recognition versus localization		pooled	2	1	3	B/W pictures and environmental sounds: congruent semantic recognition > localization task	1	3		3	2
65	108	Stevenson	2009	Table 1B AVtools > AVspeech	11		1	1	2	Hand tools in use video: inverse effectiveness (degraded AV tool > AV speech)	1	1	2	3	1
	109	Stevenson	2009	Table 1*C* (Fig. 8) AVspeech > AVtools		pooled	1	1	2	Face and speech video: inverse effectiveness (degraded AV speech > AV tool use)	1	1		1	1
66	110	Straube	2011	Table 3*A* and *B* iconic/metaphoric speech-gestures versus speech, gesture	16		2	2	4	Integration of Iconic and Metaphoric speech-gestures versus speech and gesture	1	1		1	1
67	111	Straube	2014	p939 Integration foci	16		3	0	3	Integration of iconic hand gesture-speech > unimodal speech and unimodal gesture (healthy control group)	1	1		1	1
68	112	Szycik	2009	Table 1 AV incongruent > AV congruent face+speech	11		7	2	9	Incongruent AV face-speech versus congruent AV face-speech	2	1		1	1
69	113	Tanabe	2005	Table 1*A* AV; A then V; not VA	15		10	10	20	Amorphous texture patterns and modulated white noises: Activation during learning delay period (AV)	1	2	2	2	2
	114	Tanabe	2005	Table 2A+2B (Fig. 5*A*) AV and VA		pooled	5	6	11	Amorphous texture patterns and modulated white noises: changes after feedback learning (AV and VA)	1	2	2	2	2
	115	Tanabe	2005	Table 3A+3B (Fig. 6) AV and VA; delay period		pooled	9	1	10	Amorphous texture patterns and modulated white noises: sustained activity throughout learning (AV and VA)	1	2	2	2	2
70	116	Taylor	2006	pg 8240 AV incongruent	15		1	0	1	Color photos (V), environmental sounds (A), spoken words: Incongruent (living objects)	2	0	0	0	0
	117	Taylor	2006	Figure 1*A* and *B*, Figure 1*C* and *D* (living > nonliving)		pooled	2	0	2	Color photos (V), environmental sounds and spoken words (A): Cong AV versus Incong (living objects)	1				2
71	118	Van Atteveldt	2004	Table 1*A* letters and speech sounds	16		3	1	4	Familiar letters and their speech sounds: Congruent versus not and Bimodal versus Unimodal	1			1	2
72	119	Van Atteveldt	2007	Table 2A+B (Fig. 2)	12		3	2	5	Single letters and their speech sounds (phonemes): Congruent > Incong; Passive perception, blocked and event-related design	1			1	2
	120	Van Atteveldt	2007	Table 3 (Fig. 2) passive		pooled	1	1	2	Single letters and their speech sounds (phonemes): Congruent > Incong, active perception task	1			1	2
	121	Van Atteveldt	2007	Table 4 (Fig. 6) active condition, incongruent		pooled	1	6	7	Single letters and their speech sounds (phonemes): Incongruent > Congruent	2	0	0	0	0
73	122	Van Atteveldt	2010	Table 1B STS; specific adaptation congruent > incong	16		3	1	4	Letter and speech sound pairs (vowels, consonants): Specific adaptation effects	1			1	2
74	123	Van der Wyk	2010	Table 2 AV interaction effects oval/circles+speech/nonspeech	16		3	3	6	Geometric shape modulate with speech (sentences)	1			1	1
75	124	Von Kriegstein	2006	Figure 4*B* after > before voice-face	14		0	4	4	Face and object photos with voice and other sounds: Voice-Face association learning	1	1		1	2
76	125	Watkins	2006	Figure 4 illusory multisensory interaction	11		0	2	2	Two brief tone pips leads to illusion of two screen flashes (annulus with checkerboard) when only one flash present	1	2		2	
	126	Watkins	2006	Table 1 (A enhances V in general)		pooled	5	3	8	Single brief tone pip leads to illusion of single screen flash (annulus with checkerboard) when two flashes present	1	2		2	
77	127	Watkins	2007	Figure 3 2 flashes +1 beep illusion	10		0	1	1	Two visual flashes and single audio bleep leads to the illusion of a single flash	1	2		2	
78	128	Watson	2014a	Table 1*A* AV-adaptation effect (multimodal localizer)	18		0	1	1	Videos of emotional faces and voice: multisensory localizer	1	1		1	1
	129	Watson	2014a	Table 1*C* AV-adaptation effect, cross-modal adaptation effect		pooled	0	1	1	Videos of emotional faces and voice: crossmodal adaptation effects	1	1	1	1	1
79	130	Watson	2014b	Table 1 AV > baseline (Living and nonliving)	40		3	5	8	Moving objects and videos of faces with corresponding sounds: AV > baseline	1				1
	131	Watson	2014b	Table 4*A* integrative regions (Living and nonliving)		pooled	2	2	4	Moving objects and videos of faces with corresponding sounds: Integrative regions (AV > A,V)	1				1
	132	Watson	2014b	Table 4*B* integrative regions (Living and nonliving)		pooled	0	1	1	Moving objects and videos of faces with corresponding sounds: People-selective integrative region	1	1		1	1
80	133	Werner	2010	Table 1 superadditive (AV-salience effect)	21		0	3	3	Categorize movies of actions with tools or musical instruments (degraded stimuli); AV interactions both tasks	1	1	2	2	1
	134	Werner	2010	Table 2 AV interactions predict behavior		pooled	1	2	3	Categorize movies of actions with tools or musical instruments; AV interactions predicted by behavior	1	1	2	2	1
	135	Werner	2010	Table 3*C* superadditive AV due to task		pooled	3	0	3	Categorize movies of actions with tools or musical instruments; Subadditive AV to task	1	1	2	2	1
81	136	Willems	2007	Table 3*C* and *D* mismatch hand gestures and speech	16		2	1	3	Mismatch of hand gesture (no face) and speech versus correct	2	1		1	1
82	137	Wolf	2014	Table 1 face cartoons + phonemes	16		1	1	2	Drawing of faces with emotional expressions: Supramodal effects with emotional valence	1	1	1	1	2

Notes: The first column denotes the 82 included studies, and the second column the 137 experimental paradigms of those studies. The next columns depict first author (alphabetically), the year, and abbreviated description of the data table (T) or figure (F) used, followed by number of subjects. The column labeled “Multiple experiments” indicates that the multiple experimental paradigms where subject numbers were pooled from that study for the meta-analysis, such as for the single study ALE meta-analysis depicted in Figure 3*A* (purple). The number of reported foci in the left and right hemispheres and their sum is also indicated. This is followed by a brief description of the experimental paradigm: B/W = black and white, A = audio, AV = audio-visual, V = visual, VA = visual-audio. The rightmost columns show the coding of experimental paradigms that appear in subsequent meta-analyses and Tables, with correspondence to the results illustrated in Figure 3: 0 = not used in contrast, 1 = included, 2 = included as the contrast condition, blank cell = uncertain of clear category membership and not used in that contrast condition. See text for other details.

### Activation Likelihood Estimate Analyses

The ALE analysis consists of a coordinate-based, probabilistic meta-analytic technique for assessing the colocalization of reported activations across studies (Turkeltaub et al. 2002, 2012; Eickhoff et al. 2009, 2012, 2016; Laird et al. 2009, 2010; Muller et al. 2018). Whole-brain probability maps were initially created across all the reported foci in standardized stereotaxic space (Talairach “T88,” being converted from, for example, Montreal Neurological Institute “MNI” format) using GingerALE software (Brainmap GingerALE version 2.3.6; Research Imaging Institute; http://brainmap.org). This software was also used to create probability maps, where probabilities were modeled by 3D Gaussian density distributions that took into account sample size variability by adjusting the full-width half-max (FWHM) for each study (Eickhoff et al. 2009; Eickhoff et al. 2016). For each voxel, GingerALE estimated the cumulative probabilities that at least one study reported activation for that locus for a given experimental paradigm condition. Assuming and accounting for spatial uncertainty across reports, this voxel-wise procedure generated statistically thresheld ALE maps, wherein the resulting ALE values reflected the probability of reported activation at that locus. Using a random effects model, the values were tested against the null hypothesis that activation was independently distributed across all studies in a given meta-analysis.

To determine the likely spatial convergence of reported activations across studies, activation foci coordinates from experimental paradigms were transferred manually and compiled into one spreadsheet on two separate occasions by two different investigators (coauthors). To avoid (or minimize) the potential for errors (e.g., transformation from MNI to TAL, sign errors, duplicates, omissions, etc.) an intermediate stage of data entry involved logging all the coordinates and their transformations into one spreadsheet (Appendix A) where they were coded by Table/Figure and number of subjects (Table 1), facilitating inspection and verification relative to hard copy printouts of all included studies. A third set of files (text files) were then constructed from that spreadsheet of coordinates and entered as input files for the various meta-analyses using GingerALE software. This process enabled a check-sums of number of left and right hemisphere foci and the number of subjects for all of the meta-analyses reported herein. When creating single study data set analysis ALE maps, coordinates from experimental paradigms of a given study (using the same participants in each paradigm) were pooled together, thereby avoiding potential violations of assumed subject-independence across maps, which could negatively impact the validity of the meta-analytic results (Turkeltaub et al. 2012). After pooling, there were 1285 participants (Table 1, column 6). Some participants could conceivably have been recruited in more than one study (such as from the same laboratory). However, we had no means for assessing this and assumed that these were all unique individuals. All single study data set ALE maps were thresheld at *P* < 0.05 with a voxel-level family-wise error (FWE) rate correction for multiple comparisons (Muller et al. 2018) using 10 000 Monte Carlo threshold permutations. For all “contrast” ALE meta-analysis maps, cluster-level thresholds were derived using the single study corrected FWE datasets and then further thresheld for contrast at an uncorrected *P* < 0.05, and using 10 000 permutations. Minimum cluster sizes were used to further assess rigorousness of clusters, which are included in the tables and addressed in Results.

Guided by earlier meta-analyses of hearing perception and audio-visual interaction sites, several hypothesis driven contrasts were derived as addressed in the Introduction (Lewis 2010; Brefczynski-Lewis and Lewis 2017). A minimum of 17–20 studies was generally recommended to achieve sufficient statistical power to detect smaller effects and make sure that results were not driven by single experiments (Eickhoff et al. 2016; Muller et al. 2018). However, 2 of the 10 subsets of meta-analysis were performed despite there being relatively few numbers of studies (i.e., *n* = 13 in Table 9; *n* = 9 in Table 10), and thus their outcomes would presumably only reveal the larger effect sizes and merit future study. For visualization purposes, resulting maps were initially projected onto the N27 atlas brain using AFNI software (Cox 1996) to assess and interpret results, and onto the population-averaged, landmark-, and surface-based (PALS) atlas cortical surface models (in AFNI-Talairach space) using Caret software (http://brainmap.wustl.edu) for illustration of the main findings (Van Essen et al. 2001; Van Essen 2005).

## Results

**Figure 3:**
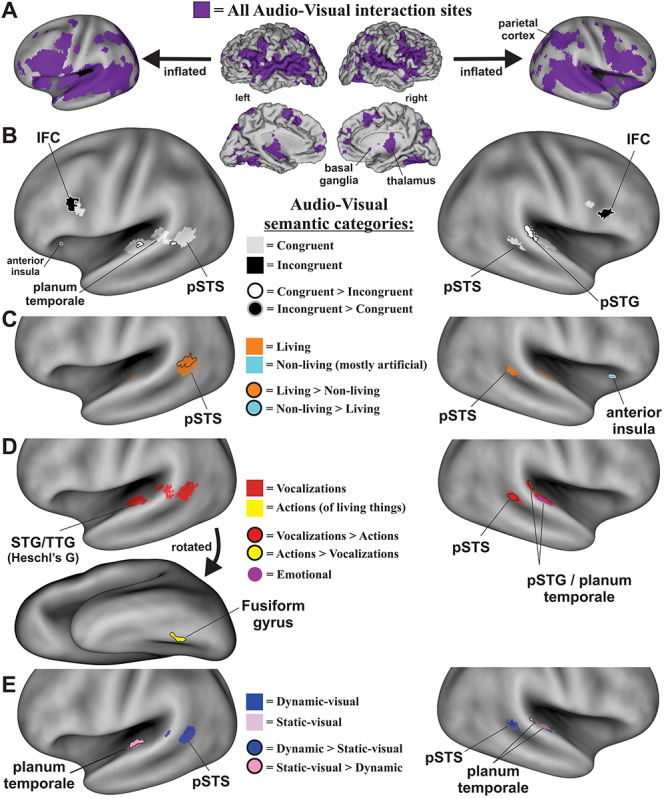
ALE maps of audio-visual interaction sites. (*A*) Cortical maps derived from all studies (Table 1; purple hues, unthresholded) to illustrate global expanses of cortices involved. Data were projected onto the fiducial (lateral and medial views) and inflated (lateral views only) PALS atlas model of cortex. (*B*) Illustration of maps derived from single study congruent paradigms (white hues) plus superimposed maps of single study incongruent audio-visual paradigms (black). Outlined foci depict ROIs surviving after direct contrasts (e.g., congruent > incongruent). (*C*) ALE map revealing single study living (orange) contrasted with single study nonliving (cyan) categories of audio-visual paradigms and outlined foci that survived after direct contrasts. (*D*) ALE maps revealing audio-visual interactions involving single study vocalizations (red) versus single study action (mostly nonvocal) living source sounds (yellow), and outlined foci that survived after direct contrasts. A single study ALE map for paradigms using emotionally valent audio-visual stimuli, predominantly involving human vocalizations, is also illustrated (violet). (*E*) ALE maps showing ROIs preferentially recruited using single study dynamic-visual (blue hues) relative to single study static-visual (pink hues) audio-visual interaction foci, and outlined foci that survived after direct contrasts. All single study ALE maps were at corrected FWE *P* < 0.05, and subsequently derived contrast maps were at uncorrected *P* < 0.05. IFC = inferior frontal cortex, pSTS = posterior superior temporal sulcus, TTG = transverse temporal gyrus. Refer to text for other details.

The database search for audio-visual experiments reporting interaction effects yielded 137 experimental paradigms from 82 published articles (Fig. 2; PRISMA flow-chart). Experiments revealing an effect of audio-visual stimuli (Table 1) included 1285 subjects (though see Materials and Methods) and 714 coordinate brain locations (376 left hemisphere, 338 right). ALE meta-analysis of all these reported foci (congruent plus incongruent audio-visual interaction effects) revealed a substantial expanse of activated brain regions (Fig. 3*A*, purple hues; projected onto both fiducial and inflated brain model images). Note that this unthresholded map revealed foci reported as demonstrating audio-visual interactions that were found to be significant in at least one of the original studies, thereby illustrating the substantial global expanse of reported brain territories involved in audio-visual interaction processing in general. This included subcortical in addition to cortical regions, such as the thalamus and basal ganglia (Fig. 3*A* insets), and cerebellum (not illustrated). However, subcortical regions are only approximately illustrated here since they did not survive threshold criteria imposed in the below single study and contrast ALE brain maps. Each study contained one or multiple experimental paradigms. For each experimental paradigm, several neurobiological subcategories of audio, visual, and/or audio-visual stimuli were identified. The subcategories are coded in Table 1 (far right columns) as either being excluded (0), included (1), included as a contrast condition (2), or deemed as uncertain for inclusion (blank cells) for use in different meta-analyses. Volumes resulting from the meta-analyses (depicted in Fig. 3) are available in Supplementary Material.

### Congruent versus Incongruent Audio-Visual Stimuli

The first set of meta-analyses examined reported activation foci specific to when audio-visual stimuli were perceived as congruent spatially, temporally, and/or semantically (Table 2; 79 studies, 117 experimental paradigms, 1235 subjects, 608 reported foci—see Table captions) versus those regions more strongly activated when the stimuli were perceived as incongruent (Table 3). Brain maps revealing activation when processing only congruent audio-visual pairings (congruent single study; corrected FWE *P* < 0.05) revealed several regions of interest (ROIs) (Fig. 3*B*, white hues; Table 4*A* coordinates), including the bilateral posterior superior temporal sulci (pSTS) that extended into the bilateral planum temporale and transverse temporal gyri (left > right), and the bilateral inferior frontal cortices (IFC). Brain maps revealing activation when processing incongruent audio-visual pairings (Fig. 3*B*, black hues; incongruent single study; corrected FWE *P* < 0.05; Table 4*B*) revealed bilateral IFC foci that were located immediately anterior to the IFC foci for congruent stimuli, plus a small left anterior insula focus.

**Table 2:** Studies included in the Congruent category for audio-visual interaction site meta-analyses

Study #	Experiment #				# Subjects	Multiple experiments	Left hem foci	Right hem foci	Number of foci	
79	117	First author	Year	Experimental code and abbreviated task	1235		320	288	608	Brief description of experimental paradigm
1	1	Adams	2002	Expt 1 Table 3 A + V (aud coords only)	12		5	1	6	A and V commonly showing subordinate > basic object name verification (words with pictures or environmental sounds)
2	2	Alink	2008	Table 1*c* spheres move to drum sounds	10		4	6	10	Visual spheres and drum sounds moving: crossmodal dynamic capture versus conflicting motion
3	3	Balk	2010	Figure 2 asynchronous versus simultaneous	14		2	1	3	Natural asynchronous versus simultaneous AV speech synchrony (included both contrasts as interaction effects)
4	4	Baumann	2007	Table 1*B* coherent V + A versus A	12		2	1	3	Visual dots 16% coherent motion and in-phase acoustic noise > stationary acoustic sound
	5	Baumann	2007	Table 2*B*		pooled	15	12	27	Moving acoustic noise and visual dots 16% in-phase coherent > random dot motion
5	6	Baumgaertner	2007	Table 3 Action > nonact sentence+video	19		3	0	3	Conjunction spoken sentences (actions > nonactions) AND videos (actions > nonactions)
6	7	Beauchamp	2004a	Figure 3*J* and *K*, Table 1 first 2 foci only	26		2	0	2	See photographs of tools, animals and hear corresponding sounds versus scrambled images and synthesized rippled sounds
7	8	Beauchamp	2004b	Expt 1 coordinates	8		1	1	2	High resolution version of 2004a study: AV tool videos versus unimodal (AV > A,V)
8	9	Belardinelli	2004	Table 1 AV semantic congruence	13		6	6	12	Colored images of tools, animals, humans and semantically congruent versus incongruent sounds
9	11	Biau	2016	Table 1*A* Interaction; speech synchronous	17		8	0	8	Hand gesture beats versus cartoon disk and speech interaction: synchronous versus asynchronous
10	12	Bischoff	2007	Table 2*A* only *P* < 0.05 included	19		2	1	3	Ventriloquism effect: gray disks and tones, synchronous (*P* < 0.05 corrected)
11	13	Blank	2013	Figure 2	19		1	0	1	Visual-speech recognition correlated with recognition performance
12	14	Bonath	2013	pg 116 congruent thalamus	18		1	0	1	Small checkerboards and tones: spatially congruent versus incongruent (thalamus)
13	16	Bonath	2014	Table 1*A* illusory versus not	20		1	5	6	Small checkerboards and tones: temporal > spatial congruence
	17	Bonath	2014	Table 1*B* synchronous > no illusion		pooled	3	0	3	Small checkerboards and tones: spatial > temporal congruence
14	18	Bushara	2001	Table 1*A* (Fig. 2) AV-Control	12		1	3	4	Tones (100 ms) and colored circles synchrony: detect Auditory then Visual presentation versus Control
	19	Bushara	2001	Table 1*B* (VA-C) five coords		pooled	2	3	5	Tones (100 ms) and colored circles synchrony: detect Visual then Auditory presentation versus Control
	20	Bushara	2001	Table 2*A* interact w/Rt Insula		pooled	2	4	6	Tones and colored circles: correlated functional connections with (and including) the right insula
15	21	Bushara	2003	Table 2*A* collide > pass, strong A-V interact	7		5	3	8	Tone and two visual bars moving: Tone synchrony induce perception they collide (AV interaction) versus pass by
16	22	Callan	2014	Table 5 AV-Audio (AV10-A10)-(AV6-A6)	16		4	4	8	Multisensory enhancement to visual speech in noise correlated with behavioral results
	23	Callan	2014	Table 6 AV—Visual only		pooled	1	1	2	Multisensory enhancement to visual speech audio-visual versus visual only
17	24	Calvert	1999	Table 1 (Fig. 1)	5		3	4	7	View image of lower face and hear numbers 1 through 10 versus unimodal conditions (AV > Photos, Auditory)
18	25	Calvert	2000	Figure 2 superadd+subadd AVspeech	10		1	0	1	Speech and lower face: supra-additive plus subadditive effects (AV-congruent > A,V > AV-incongruent)
	26	Calvert	2000	Table 1 supradd AVspeech		pooled	4	5	9	Speech and lower face: supra-additive AV enhancement
19	28	Calvert	2001	Table 2 superadditive and resp depression	10		4	11	15	B/W visual checkerboard reversing and white noise bursts: Synchronous versus not; supradditive and response depression
	29	Calvert	2001	Table 3*A* superadditive only		pooled	6	4	10	B/W visual checkerboard reversing and white noise bursts: Synchronous versus not; supradditive only
	30	Calvert	2001	Table 3*B* response depression only		pooled	3	4	7	B/W visual checkerboard reversing and white noise bursts: Synchronous versus not; response depression only
20	31	Calvert	2003	Table 2*A* (Fig. 3 blue)	8		13	8	21	Speech and lower face: Moving dynamic speech (phonemes) versus stilled speech frames
21	32	DeHaas	2013	Table 1*A* AVcong—Visual	15		3	3	6	Video clips of natural scenes (animals, humans): AV congruent versus Visual
22	34	Erickson	2014	Table 1*A* Congruent AV speech	10		2	2	4	McGurk effect (phonemes): congruent AV speech: AV > A and AV > V
	35	Erickson	2014	Table 1*B* McGurk speech		pooled	2	0	2	McGurk speech effect (phonemes)
23	36	Ethofer	2013	Table 1C emotion	23		1	2	3	Audiovisual emotional face-voice integration
24	37	Gonzalo	2000	Table 1 AV > AVincon music and Chinese ideograms	14		1	1	2	Learn novel Kanji characters and musical chords, activity increases over time for consistent AV pairings
	39	Gonzalo	2000	Table 3 AV consistent versus Aud		pooled	1	1	2	Learn novel Kanji characters and musical chords, learn consistent (vs inconsistent) pairings versus auditory only
25	41	Green	2009	Table 4*A* Congruent gesture-speech > gesture or speech	16	pooled	1	0	1	Congruent gesture-speech versus gesture with unfamiliar speech and with familiar speech
26	42	Hagan	2013	Table 1 AV emotion, novel over time	18		5	3	8	Affective audio-visual speech: congruent AV emotion versus A, V; unique ROIs over time (MEG)
27	44	Hasegawa	2004	Table 1A (well trained piano) AV induced by V-only	26		12	6	18	Piano playing: well trained pianists, mapping hand movements to sequences of sound
28	45	Hashimoto	2004	Table 1*G* (Fig 4B, red) Learning Hangul letters to sounds	12		2	1	3	Unfamiliar Hangul letters and nonsense words, learn speech versus tone/noise pairings
29	46	He	2015	Table 3C AV speech foreign (left MTG focus)	20		1	0	1	Intrinsically meaningful gestures with German speech: Gesture-German > Gesture-Russian, German speech only
30	47	He	2018	Table 2, GSI, left MTG, gestures and speech integration	20		1	0	1	Gesture-speech integration: Bimodal speech-gesture versus unimodal gesture with foreign speech and versus unimodal speech
31	49	Hein	2007	Figure 2*B* AV-artificial/nonliving	18		0	1	1	B/W images of artificial objects (“fribbles”) and animal vocalizations versus unimodal A, V
	50	Hein	2007	Figure 2*C* pSTS, pSTG, mSTG AV-cong		pooled	0	3	3	Familiar animal images and correct vocalizations (dog: woof-woof)
	52	Hein	2007	Figure 3*B* Foci 2, 3, 4 (blue) artificial/nonliving		pooled	3	0	3	Visual “Fribbles” and backward/underwater distorted animal sounds, learn pairings (blue foci 2,3,4)
	53	Hein	2007	Figure 3*C* congruent living (green)		pooled	3	0	3	Familiar congruent living versus artificial AV object features and animal sounds (green foci 7, 8, 10)
32	54	Hocking	2008	pg 2444 verbal	18		2	0	2	(pSTS mask) Color photos, written names, auditory names, environmental sounds conceptually matched “amodal”
33	56	Hove	2013	pg 316 AV interaction putamen	14		0	1	1	Interaction between (beep > flash) versus (siren > moving bar); left putamen focus
34	57	James	2003	Figure 2	12		0	1	1	Activation by visual objects (“Greebles”) associated with auditory features (e.g., buzzes, screeches); (STG)
35	58	James	2011	Table 1*A* bimodal (vs. scrambled)	12		4	2	6	Video of human manual actions (e.g., sawing): Auditory and Visual intact versus scrambled, AV event selectivity
36	59	Jessen	2015	Table 1*A* emotion > neutral AV enhanced	17		1	1	2	Emotional multisensory whole body and voice expressions: AV emotion (anger and fear) > neutral expressions
	60	Jessen	2015	Table 1*D* fear > neutral AV enhanced		pooled	2	1	3	Emotional multisensory whole body and voice expressions: AV fear > neutral expressions
37	61	Jola	2013	Table 1*C* AVcondition dance	12		3	3	6	Viewing unfamiliar dance performance (tells a story by gesture) with versus without music: using intersubject correlation
38	62	Kim	2015	Table 2*A* AV > C speech semantic match	15		2	0	2	Moving audio-visual speech perception versus white noise and unopened mouth movements
39	63	Kircher	2009	Figure 3*B*: gesture related activation increase	14		3	1	4	Bimodal gesture-speech versus gesture and versus speech
40	64	Kreifelts	2007	Table 1 voice-face emotion	24		1	2	3	Facial expression and intonated spoken words, judge emotion expressed (AV > A,V; *P* < 0.05 only)
	65	Kreifelts	2007	Table 5 AV increase effective connectivity		pooled	2	4	6	Increased effectiveness connectivity with pSTS and thalamus during AV integration of nonverbal emotional information
41	66	Lewis	2000	Table 1	7		2	3	5	Compare speed of tone sweeps to visual dot coherent motion: Bimodal versus unimodal
42	67	Matchin	2014	Table 1 AV > Aud only (McGurk)	20		2	7	9	McGurk audio-visual speech: AV > A only
	68	Matchin	2014	Table 2 AV > Video only		pooled	9	6	15	McGurk audio-visual speech: AV > V only
43	70	McNamara	2008	Table (BA44 and IPL)	12		2	2	4	Videos of meaningless hand gestures and synthetic tone sounds: Increases in functional connectivity with learning
44	71	Meyer	2007	Table 3 paired A + V versus null	16		3	3	6	Paired screen red flashes with phone ring: paired V (conditioned stimulus) and A (unconditioned) versus null events
	72	Meyer	2007	Table 4 CS+, learned AV association with V-only		pooled	4	6	10	Paired screen flashes with phone ring: View flashes after postconditioned versus null events
45	73	Muller	2012	Supplementary Table 1 effective connectivity changes	27		4	3	7	Emotional facial expression (groaning, laughing) AV integration and gating of information
47	75	Naghavi	2007	Figure 1C	23		0	3	3	B/W pictures (animals, tools, instruments, vehicles) and their sounds: congruent versus incongruent
48	77	Naghavi	2011	Figure 2*B* congruent > incongruent	30		0	1	1	B/W drawings of objects (living and non) and natural sounds (barking, piano): congruent > incongruent encoding
49	79	Nath	2012	pg 784	14		1	0	1	McGurk effect (phonemes): congruent AV speech correlated with behavioral percept
50	80	Naumer	2008	Figure 2 Table 1*A* max contrast	18		8	6	14	Images of “Fribbles” and learned artificial sounds (underwater animal vocals): posttraining versus max contrast
	81	Naumer	2008	Figure 3 Table 1*B* pre–post		pooled	5	6	11	Images of “Fribbles” and learned corresponding artificial sounds: Post- versus pretraining session
51	83	Naumer	2011	Figure 3*C*	10		1	0	1	Photographs of objects (living and non) and related natural sounds
52	85	Noppeny	2008	Table 3 AV congruent sounds/words	17		4	0	4	Speech sound recognition through AV priming, environmental sounds and spoken words: Congruent > incongruent
53	86	Ogawa	2013a	Table 1 (pg 162 data)	13		1	0	1	AV congruency of pure tone and white dots moving on screen (area left V3A)
54	87	Ogawa	2013b	Table 1 3D > 2D and surround > monaural effects	16		3	4	7	Cinematic 3D > 2D video and surround sound > monaural while watching a movie (“The Three Musketeers”)
55	88	Okada	2013	Table 1 AV > A	20		5	4	9	Video of AV > A speech only
56	89	Olson	2002	Table 1*A* synchronized AV > static Vis-only	10		7	4	11	Whole face video and heard words: Synchronized AV versus static V
	90	Olson	2002	Table 1*C* synchronized AV > desynchronized AV speech		pooled	2	0	2	Whole face video and heard words: Synchronized versus desynchronized
57	91	Plank	2012	pg 803 AV congruent effect	15		0	1	1	AV spatially congruent > semantically matching images of natural objects and associated sounds (right STG)
	92	Plank	2012	Table 2*A* spatially congruent-baseline		pooled	5	5	10	Images of natural objects and associated sounds, spatially congruent versus baseline
58	93	Raij	2000	Table 1*B* letters and speech sounds	9		2	3	5	Integration of visual letters and corresponding auditory phonetic expressions (MEG study) AV versus (A + V)
59	94	Regenbogen	2017	Table 2*A* degraded > clear Multisensory versus unimodal input	29		5	6	11	Degraded > clear AV versus both visual and auditory unimodal visual real-world object-in-action recognition
60	95	Robins	2008	Table 2 (Fig. 2) AV integration (AV > A and AV > V)	10		2	1	3	Face speaking sentences: angry, fearful, happy, neutral (AV > A,V)
	96	Robins	2008	Table 4*A* (Fig. 5) AV integration and emotion	5		1	4	5	AV faces and spoken sentences expressing fear or neutral valence: AV integration (AV > A,V conditions)
	97	Robins	2008	Table 4*B* emotion effects		pooled	2	0	2	AV faces and spoken sentences expressing fear or neutral valence: Emotional AV-fear > AV-neutral
	98	Robins	2008	Table 4*C* (Fig. 5) fearful AV integration		pooled	1	5	6	AV faces and spoken sentences expressing fear or neutral valence: Fearful-only AV integration
	99	Robins	2008	Table 4*D* AV-only emotion		pooled	1	3	4	AV faces and spoken sentences expressing fear or neutral valence: AV-only emotion
61	100	Scheef	2009	Table 1 cartoon jump + boing	16		1	2	3	Video of cartoon person jumping and “sonification” of a tone, learn correlated pairings: AV-V and AV-A conjunction
62	101	Schmid	2011	Table 2E A effect V (Living and nonliving, pictures)	12		3	4	7	Environmental sounds and matching pictures: reduced activity by A
	102	Schmid	2011	Table 2*F* V competition effect A (reduced activity by a visual object)		pooled	2	2	4	Environmental sounds and matching pictures: reduced activity by V
	103	Schmid	2011	Table 2*G* AV crossmodal interaction x auditory attention		pooled	2	3	5	Environmental sounds and matching pictures: cross-modal interaction and auditory attention
63	104	Sekiyama	2003	Table 3 (fMRI nAV-AV)	8		1	0	1	AV speech, McGurk effect with phonemes (ba, da, ga) and noise modulation: noise-AV > AV (fMRI)
	105	Sekiyama	2003	Table 4 (PET nAV-AV)		pooled	1	3	4	AV speech, McGurk effect with phonemes (ba, da, ga) and noise modulation: noise-AV > AV (PET)
64	106	Sestieri	2006	Table 1 (Fig. 3), AV location match versus semantic	10		2	5	7	B/W images (animal, weapons) and environmental sounds: Match location > recognition
	107	Sestieri	2006	Table 2 AV semantic recognition versus localization		pooled	2	1	3	B/W pictures and environmental sounds: congruent semantic recognition > localization task
65	108	Stevenson	2009	Table 1B 2 AVtools > AVspeech	11		1	1	2	Hand tools in use video: inverse effectiveness (degraded AV tool > AV speech)
	109	Stevenson	2009	Table 1*C* (Fig. 8) AVspeech > AVtools		pooled	1	1	2	Face and speech video: inverse effectiveness (degraded AV speech > AV tool use)
66	110	Straube	2011	Table 3*A* and *B* iconic/metaphoric speech-gestures versus speech, gesture	16		2	2	4	Integration of Iconic and Metaphoric speech-gestures versus speech and gesture
67	111	Straube	2014	p939 Integration foci	16		3	0	3	Integration of iconic hand gesture-speech > unimodal speech and unimodal gesture (healthy control group)
69	113	Tanabe	2005	Table 1*A* AV; A then V; not VA	15		10	10	20	Amorphous texture patterns and modulated white noises: Activation during learning delay period (AV)
	114	Tanabe	2005	Table 2A+2B (Fig. 5a) AV and VA		pooled	5	6	11	Amorphous texture patterns and modulated white noises: changes after feedback learning (AV and VA)
	115	Tanabe	2005	Table 3A+3B (Fig. 6) AV and VA; delay period		pooled	9	1	10	Amorphous texture patterns and modulated white noises: sustained activity throughout learning (AV and VA)
70	117	Taylor	2006	Figure 1*A* and *B*, Figure 1*C* and *D* (living > nonliving)	15		2	0	2	Color photos (V), environmental sounds and spoken words (A): Congruent AV versus Incongruent (living objects)
71	118	Van Atteveldt	2004	Table 1*a* letters and speech sounds	16		3	1	4	Familiar letters and their speech sounds: Congruent versus not and Bimodal versus Unimodal
72	119	Van Atteveldt	2007	Table 2A+B (Fig. 2)	12		3	2	5	Single letters and their speech sounds (phonemes): Congruent > Incong; Passive perception, blocked and event-related design
	120	Van Atteveldt	2007	Table 3 (Fig. 2) passive		pooled	1	1	2	Single letters and their speech sounds (phonemes): Congruent > Incong, active perception task
73	122	Van Atteveldt	2010	Table 1*B* STS; specific adaptation congruent > incong	16		3	1	4	Letter and speech sound pairs (vowels, consonants): Specific adaptation effects
74	123	Van der Wyk	2010	Table 2 AV interaction effects oval/circles +speech/nonspeech	16		3	3	6	Geometric shape modulate with speech (sentences)
75	124	Von Kriegstein	2006	Figure 4*B* after > before voice-face	14		0	4	4	Face and object photos with voice and other sounds: Voice-Face association learning
76	125	Watkins	2006	Figure 4 illusory multisensory interaction	11		0	2	2	Two brief tone pips leads to illusion of two screen flashes (annulus with checkerboard) when only one flash present
	126	Watkins	2006	Table 1 (A enhances V in general)		pooled	5	3	8	Single brief tone pip leads to illusion of single screen flash (annulus with checkerboard) when two flashes present
77	127	Watkins	2007	Figure 3 2 flashes +1 beep illusion	10		0	1	1	Two visual flashes and single audio bleep leads to the illusion of a single flash
78	128	Watson	2014a	Table 1*A* AV-adaptation effect (multimodal localizer)	18		0	1	1	Videos of emotional faces and voice: multisensory localizer
	129	Watson	2014a	Table 1*C* AV-adaptation effect, cross-modal adaptation effect		pooled	0	1	1	Videos of emotional faces and voice: crossmodal adaptation effects
79	130	Watson	2014b	Table 1 AV > baseline (Living and nonliving)	40		3	5	8	Moving objects and videos of faces with corresponding sounds: AV > baseline
	131	Watson	2014b	Table 4*A* integrative regions (Living and nonliving)		pooled	2	2	4	Moving objects and videos of faces with corresponding sounds: Integrative regions (AV > A,V)
	132	Watson	2014b	Table 4*B* integrative regions (Living and nonliving)		pooled	0	1	1	Moving objects and videos of faces with corresponding sounds: People-selective integrative region
80	133	Werner	2010	Table 1 superadditive (AV-salience effect)	21		0	3	3	Categorize movies of actions with tools or musical instruments (degraded stimuli); AV interactions both tasks
	134	Werner	2010	Table 2 AV interactions predict behavior		pooled	1	2	3	Categorize movies of actions with tools or musical instruments; AV interactions predicted by behavior
	135	Werner	2010	Table 3*C* superadditive AV due to task		pooled	3	0	3	Categorize movies of actions with tools or musical instruments; Subadditive AV to task
82	137	Wolf	2014	Table 1 face cartoons + phonemes	16		1	1	2	Drawing of faces with emotional expressions: Supramodal effects with emotional valence

Note: This meta-analysis included 79 of the studies with 117 experiments (first and second column). The column “multiple experiments” indicates paradigms where the same set of participants were included, and so all coordinates were pooled together as one study to avoid biases related to violation the assumption of subject independence (refer to Materials and Methods). Results of Congruent meta-analyses are shown in Figure 3*B* white hues. Refer to Table 1 and text for other details.

**Table 3:** Studies included in the Incongruent category for audio-visual interaction site meta-analyses

Study #	Experiment #				# Subjects	Multiple experiments	Left hem foci	Right hem foci	Number of foci	
18	19	First author	Year	Experimental code and abbreviated task	307		55	50	105	Brief description of experimental paradigm
8	10	Belardinelli	2004	Table 2 AV semantic incongruent	13		2	3	5	Colored images of tools, animals, humans and semantically incongruent versus congruent sounds
12	15	Bonath	2013	pg 116 incongruent	18		1	1	2	Small checkerboards and tones: spatially incongruent versus congruent (thalamus)
18	27	Calvert	2000	Table 2 incongruent subadditive AVspeech	10		3	3	6	Speech and lower face: subadditive AV response to incongruent AV inputs
21	33	DeHaas	2013	Table 1*B* V- AV incongruent	15		2	0	2	Video clips of natural scenes (animals, humans): Visual versus AV incongruent
24	38	Gonzalo	2000	Table 2 inconsistent AV	14		4	4	8	Learn novel Kanji characters and musical chords, activity increases over time to inconsistent pairings
25	40	Green	2009	Table 1 incongruent > congruent gesture-speech	16		4	5	9	Incongruent versus congruent gesture-speech
26	43	Hagan	2013	Table 2 AV emotion incongruent	18		1	5	6	Affective audio-visual speech: incongruent AV emotion versus A, V; unique ROIs over time (MEG)
31	48	Hein	2007	Figure 2*A* AV incongruent	18		0	2	2	Familiar animal images and incorrect (incongruent) vocalizations (dog: meow) versus correct pairs
	51	Hein	2007	Figure 3*A* incongruent			4	0	4	AV familiar incongruent versus unfamiliar artificial (red foci 1,5,6,9)
32	55	Hocking	2008	Table 3 incongruent simultaneous matching	18		8	10	18	Incongruent sequential AV pairs (e.g., see drum, hear bagpipes) versus congruent pairs
42	69	Matchin	2014	Table 3 MM > AV McGurk	20		7	4	11	McGurk Mismatch > AV speech integration
46	74	Murase	2008	Figure 4 discordant > concordant AVinteraction	28		1	0	1	Audiovisual speech (syllables) showing activity to discordant versus concordant stimuli: left mid-STS
48	78	Naghavi	2011	Figure 2*C* incongruent > congruent	30		1	1	2	B/W drawings of objects (living and non) and natural sounds (barking, piano): incongruent > congruent encoding
50	82	Naumer	2008	Figure 4 Table 2	18		1	1	2	Learn of “Freebles” and distorted sounds as incongruent > congruent pairs
52	84	Noppeny	2008	Table 2 AV incongruent > congruent	17		5	2	7	Speech sound recognition through AV priming, environmental sounds and spoken words: incongruent > congruent
68	112	Szycik	2009	Table 1 AV incongruent > AV congruent face+speech	11		7	2	9	Incongruent AV face-speech versus congruent AV face-speech
70	116	Taylor	2006	pg 8240 AV incongruent	15		1	0	1	Color photos (V), environmental sounds (A), spoken words (W): Incongruent (living objects)
72	121	Van Atteveldt	2007	Table 4 (Fig. 6) active condition, incongruent	12		1	6	7	Single letters and their speech sounds (phonemes): incongruent > congruent
81	136	Willems	2007	Table 3*C* and *D* mismatch hand gestures and speech	16		2	1	3	Mismatch of hand gesture (no face) and speech versus correct

Note: Results shown in Figure 3*B* black hues. Refer to Tables 1 and 2 for other details.

**Table 4:** Locations of significant clusters from the meta-analyses involving Congruent and Incongruent audio-visual paradigms

Condition	Region	Major contributing studies	*x*	*y*	*z*	Volume	ALE value
A. Congruent audio-visual stimuli single study							
1	L Posterior Superior Temporal Sulcus/Gyrus	32 (3–4,6,7,10,13,18,19,22,27,31, 32,40,42,45,49,50,54,57,60,63,65–67,69,71–74,76.79,82)	−51	−36	9	4824	0.055
2	R Posterior Superior Temporal Sulcus (pSTS)	27 (4,7,13,16,20,23,31,34,40,42,45, 50,54,55, 57,60,60,65,66,71, 72,73,74,78–80)	51	−29	10	4064	0.045
3	L Inferior Frontal Cortex (posterior IFC)	4 (50,52,78,80)	−42	7	25	312	0.035
4	R Inferior Frontal Cortex (posteriorIFC)	3 (27,44,50)	46	6	31	216	0.034
B. Incongruent audio-visual stimuli single study							
1	R Middle Frontal Gyrus/anterior IFC	3 (31,52,68)	45	14	25	320	0.024
2	L Middle Frontal Gyrus/anterior IFC	3 (8,52,72)	−40	11	29	216	0.022
C. Contrast Congruent > Incongruent audio-visual stimuli							
1	R Posterior Superior Temporal Gyrus	8 (20,40,55,60,65,66)	52	−33	13	1112	2.820
2	L Posterior Superior Temporal Gyrus		−51	−42	8	168	2.149
3	L Posterior Superior Temporal Gyrus		−49	−25	5	72	1.862
D. Contrast Incongruent > Congruent audio-visual stimuli							
1	R Inferior Frontal Cortes (Middle Frontal Gyrus)	4 (31,52,68,72)	45	13	25	416	3.540
2	L Inferior Frontal Cortex (area 9)	2 (8,72)	−41	12	28	392	2.911
3	L Inferior Frontal Cortex (area 13)	2 (42,52)	−32	20	4	56	1.932

Note: Also indicated are major contributing studies to the ALE meta-analysis clusters, weighted centers of mass (*x*, *y*, and *z*) in Talairach coordinates, brain volumes (mm^3^), and ALE values. (*A*) Single study Congruent clusters, and (*B*) single study Incongruent clusters (both corrected FWE *P* < 0.05); plus contrast meta-analyses maps revealing (***C***) Congruent > Incongruent and (*D*) Incongruent > Congruent audio-visual interaction sites (both uncorrected *P* < 0.05). The coordinates correspond to foci illustrated in Figure 3*B* (black and white hues). Refer to text for other details (from Tables 2 and 3).

A contrast meta-analysis of congruent > incongruent audio-visual stimuli (Fig. 3*B*, white with black outlines; Table 4*C*, uncorrected *P* < 0.05) revealed significant involvement of the left and right posterior temporal gyri (pSTG) and pSTS regions. Conversely, a contrast map of brain regions showing significant preferential involvement in processing incongruent > congruent audio-visual stimuli (Fig. 3*B*, black with white outlines; Table 4*D*, uncorrected *P* < 0.05) included bilateral IFC, which extended along inferior portions of the middle frontal gyri in locations immediately anterior to those resulting from the congruent > incongruent contrast. Because both contrast ALE maps revealed functionally dissociated ROIs, these results are herein regarded as providing evidence for a “double-dissociation” of processing along this dimension.

### Living versus Nonliving Audio-Visual Stimuli

A major categorical distinction in the neurobiological organization mediating auditory perception is that for sounds produced by living versus nonliving sources (Fig. 1). This potential categorical processing boundary was tested in the multisensory realm by comparing reported activation foci from audio-visual interaction paradigms that involved living versus nonliving sources. The living category paradigms included visual and/or sound-source stimuli such as talking faces, hand/arm gesture with speech, body movements, tool use, and nonhuman animals (Table 5; see brief descriptions). A single study ALE meta-analysis of experimental paradigms using living stimuli revealed portions of the bilateral pSTS/pSTG regions (Fig. 3*C*, orange hues; Table 7*A*, corrected FWE *P* < 0.05). The nonliving visual and sound-source stimuli (Table 6) predominantly included artificial, as opposed to natural, audio-visual events such as flashing checkerboards, coherent dot motion, geometric objects (plus a study depicting natural environmental events), which were paired with sounds such as tones, sirens, or mechanical sounds produced by inanimate sources. A single study ALE meta-analysis of experiments using nonliving stimuli (mostly artificial stimuli) revealed the right anterior insula as a region significantly recruited (Fig. 3*C*, cyan contained within the white outline; Table 7*B*, corrected FWE *P* < 0.05: also see contrast below).

**Table 5:** Studies included in the Living category for audio-visual interaction site meta-analyses

Study #	Experiment #				# Subjects	Multiple experiments	Left hem foci	Right hem foci	Number of foci	
43	58	First author	Year	Experimental code and abbreviated task	699		145	126	271	Brief description of experimental paradigm
5	6	Baumgaertner	2007	Table 3 Action > nonact sentence+video	19		3	0	3	Conjunction spoken sentences (actions>nonactions) AND videos (actions > nonactions)
6	7	Beauchamp	2004a	Figure 3*J* and *K*, Table 1 first 2 foci only	26		2	0	2	See photographs of tools, animals and hear corresponding sounds versus scrambled images and synthesized rippled sounds
7	8	Beauchamp	2004b	Expt 1 coordinates	8		1	1	2	High resolution version of 2004a study: AV tool videos versus unimodal (AV > A,V)
8	9	Belardinelli	2004	Table 1 AV semantic congruence	13		6	6	12	Colored images of tools, animals, humans and semantically congruent versus incongruent sounds
9	11	Biau	2016	Table 1*A* Interaction; speech synchronous	17		8	0	8	Hand gesture beats versus cartoon disk and speech interaction: synchronous versus asynchronous
11	13	Blank	2013	Figure 2	19		1	0	1	Visual-speech recognition correlated with recognition performance
16	22	Callan	2014	Table 5 AV-Audio (AV10-A10)-(AV6-A6)	16		4	4	8	Multisensory enhancement to visual speech in noise correlated with behavioral results
	23	Callan	2014	Table 6 AV—Visual only		pooled	1	1	2	Multisensory enhancement to visual speech audio-visual versus visual only
17	24	Calvert	1999	Table 1 (Fig. 1)	5		3	4	7	View image of lower face and hear numbers 1 through 10 versus unimodal conditions (AV > Photos, Auditory)
18	25	Calvert	2000	Figure 2 superadditive+subadditive Avspeech	10		1	0	1	Speech and lower face: supra-additive plus subadditive effects (AV-congruent > A,V > AV-incongruent)
	26	Calvert	2000	Table 1 supradditive AVspeech		pooled	4	5	9	Speech and lower face: supra-additive AV enhancement
20	31	Calvert	2003	Table 2*A* (Fig. 3 blue)	8		13	8	21	Speech and lower face: Moving dynamic speech (phonemes) versus stilled speech frames
21	32	DeHaas	2013	Table 1*A* AVcong—Visual	15		3	3	6	Video clips of natural scenes (animals, humans): AV congruent versus Visual
22	34	Erickson	2014	Table 1*A* Congruent AV speech	10		2	2	4	McGurk effect (phonemes): congruent AV speech: AV > A and AV > V
	35	Erickson	2014	Table 1*B* McGurk speech		pooled	2	0	2	McGurk speech effect (phonemes)
23	36	Ethofer	2013	Table 1*C* emotion	23		1	2	3	Audiovisual emotional face-voice integration
25	41	Green	2009	Table 4*A* Congruent gesture-speech > gesture or speech	16		1	0	1	Congruent gesture-speech versus gesture with unfamiliar speech and with familiar speech
27	44	Hasegawa	2004	Table 1A (well trained piano) AV induced by V-only	26		12	6	18	Piano playing: well trained pianists, mapping hand movements to sequences of sound
29	46	He	2015	Table 3*C* AV speech foreign (left MTG focus)	20		1	0	1	Intrinsically meaningful gestures with German speech: Gesture-German > Gesture-Russian, German speech only
30	47	He	2018	Table 2 gestures and speech integration (left MTG)	20		1	0	1	Gesture-speech integration: Bimodal speech-gesture versus unimodal gesture with foreign speech and versus unimodal speech
31	50	Hein	2007	Figure 2*C* pSTS, pSTG, mSTG AV-cong	18		0	3	3	Familiar animal images and correct vocalizations (dog: woof-woof)
32	54	Hocking	2008	pg 2444 verbal	18		2	0	2	(pSTS mask) Color photos, written names, auditory names, environmental sounds conceptually matched “amodal”
35	58	James	2011	Table 1*A* bimodal (vs. scrambled)	12		4	2	6	Video of human manual actions (e.g., sawing): Auditory and Visual intact versus scrambled, AV event selectivity
36	59	Jessen	2015	Table 1*A* emotion > neutral AV enhanced	17		1	1	2	Emotional multisensory whole body and voice expressions: AV emotion (anger and fear) > neutral expressions
	60	Jessen	2015	Table 1*D* fear > neutral AV enhanced		pooled	2	1	3	Emotional multisensory whole body and voice expressions: AV fear > neutral expressions
37	61	Jola	2013	Table 1*C* AVcondition dance	12		3	3	6	Viewing unfamiliar dance performance (tells a story by gesture) with versus without music: using intersubject correlation
38	62	Kim	2015	Table 2*A* AV > C speech semantic match	15		2	0	2	Moving audio-visual speech perception versus white noise and unopened mouth movements
39	63	Kircher	2009	Figure 3*B* gesture-related activation increase	14		3	1	4	Bimodal gesture-speech versus gesture and versus speech
40	64	Kreifelts	2007	Table 1 voice-face emotion	24		1	2	3	Facial expression and intonated spoken words, judge emotion expressed (AV > A,V; *P* < 0.05 only)
	65	Kreifelts	2007	Table 5 AV increase effective connectivity		pooled	2	4	6	Increased effectiveness connectivity with pSTS and thalamus during AV integration of nonverbal emotional information
42	67	Matchin	2014	Table 1 AV > Aud only (McGurk)	20		2	7	9	McGurk audio-visual speech: AV > A only
	68	Matchin	2014	Table 2 AV > Video only		pooled	9	6	15	McGurk audio-visual speech: AV > V only
45	73	Muller	2012	Table S1 effective connectivity changes	27		4	3	7	Emotional facial expression (groaning, laughing) AV integration and gating of information
49	79	Nath	2012	pg 784	14		1	0	1	McGurk effect (phonemes): congruent AV speech correlated with behavioral percept
54	87	Ogawa	2013b	Table 1 3D > 2D and surround > monaural effects	16		3	4	7	Cinematic 3D > 2D video and surround sound > monaural while watching a movie (“The Three Musketeers”)
55	88	Okada	2013	Table 1 AV > A	20		5	4	9	Video of AV > A speech only
56	89	Olson	2002	Table 1*A* synchronized AV > static Vis-only	10		7	4	11	Whole face video and heard words: Synchronized AV versus static V
	90	Olson	2002	Table 1*C* synchronized AV > desynchronized AV speech		pooled	2	0	2	Whole face video and heard words: Synchronized versus desynchronized
60	96	Robins	2008	Table 4*A* (Fig. 5) AV integration and emotion	5		1	4	5	AV faces and spoken sentences expressing fear or neutral valence: AV integration (AV > A,V conditions)
	97	Robins	2008	Table 4*B* emotion effects		pooled	2	0	2	AV faces and spoken sentences expressing fear or neutral valence: Emotional AV-fear > AV-neutral
	98	Robins	2008	Table 4*C* (Fig. 5) fearful AV integration		pooled	1	5	6	AV faces and spoken sentences expressing fear or neutral valence: Fearful-only AV integration
	99	Robins	2008	Table 4D AV-only emotion		pooled	1	3	4	AV faces and spoken sentences expressing fear or neutral valence: AV-only emotion
61	100	Scheef	2009	Table 1 cartoon jump + boing	16		1	2	3	Video of cartoon person jumping and “sonification” of a tone, learn correlated pairings: AV-V and AV-A conjunction
63	104	Sekiyama	2003	Table 3 (fMRI nAV-AV)	8		1	0	1	AV speech, McGurk effect with phonemes (ba, da, ga) and noise modulation: noise-AV > AV (fMRI)
	105	Sekiyama	2003	Table 4 (PET nAV-AV)		pooled	1	3	4	AV speech, McGurk effect with phonemes (ba, da, ga) and noise modulation: noise-AV > AV (PET)
64	106	Sestieri	2006	Table 1 (Fig. 3), AV location match versus semantic	10		2	5	7	B/W images (animal, weapons) and environmental sounds: Match location > recognition
65	108	Stevenson	2009	Table 1*B* AVtools > AVspeech	11		1	1	2	Hand tools in use video: inverse effectiveness (degraded AV tool > AV speech)
	109	Stevenson	2009	Table 1*C* (Fig. 8) AVspeech > AVtools		pooled	1	1	2	Face and speech video: inverse effectiveness (degraded AV speech > AV tool use)
66	110	Straube	2011	Table 3*A* and *B* iconic/metaphoric speech-gestures versus speech, gesture	16		2	2	4	Integration of Iconic and Metaphoric speech-gestures versus speech and gesture
67	111	Straube	2014	p939 Integration foci	16		3	0	3	Integration of iconic hand gesture-speech > unimodal speech and unimodal gesture (healthy control group)
75	124	Von Kriegstein	2006	Figure 4*B* after > before voice-face	14		0	4	4	Face and object photos with voice and other sounds: Voice-Face association learning
78	128	Watson	2014a	Table 1*A* AV-adaptation effect (multimodal localizer)	18		0	1	1	Videos of emotional faces and voice: multisensory localizer
	129	Watson	2014a	Table 1*C* AV-adaptation effect, cross-modal adaptation effect		pooled	0	1	1	Videos of emotional faces and voice: crossmodal adaptation effects
79	132	Watson	2014b	Table 4*B* integrative regions (living and nonliving)	40		0	1	1	Moving objects and videos of faces with corresponding sounds: People-selective integrative region
80	133	Werner	2010	Table 1 superadditive (AV-salience effect)	21		0	3	3	Categorize movies of actions with tools or musical instruments (degraded stimuli); AV interactions both tasks
	134	Werner	2010	Table 2 AV interactions predict behavior		pooled	1	2	3	Categorize movies of actions with tools or musical instruments; AV interactions predicted by behavior
	135	Werner	2010	Table 3*C* superadditive AV due to task		pooled	3	0	3	Categorize movies of actions with tools or musical instruments; Subadditive AV to task
82	137	Wolf	2014	Table 1 face cartoons + phonemes	16		1	1	2	Drawing of faces with emotional expressions: Supramodal effects with emotional valence

Note: Results shown in Figure 3*C* orange hues. Refer to Tables 1 and 2 and text for other details.

**Table 6:** Studies included in the Nonliving category for audio-visual interaction site meta-analyses

Study #	Experiment #				# Subjects	Multiple experiments	Left hem foci	Right hem foci	Number of foci	
15	25	First author	Year	Experimental code and abbreviated task	187		93	93	186	Brief description of experimental paradigm
1	1	Adams	2002	Expt 1 Table 3 A + V (aud coords only)	12		5	1	6	A and V commonly showing subordinate > basic object name verification (words with pictures or environmental sounds)
2	2	Alink	2008	Table 1*c* spheres move to drum sounds	10		4	6	10	Visual spheres and drum sounds moving: crossmodal dynamic capture versus conflicting motion
4	4	Baumann	2007	Table 1*B* coherent V + A versus A	12		2	1	3	Visual dots 16% coherent motion and in-phase acoustic noise > stationary acoustic sound
	5	Baumann	2007	Table 2*B*		pooled	15	12	27	Moving acoustic noise and visual dots 16% in-phase coherent > random dot motion
12	14	Bonath	2013	pg 116 congruent thalamus	18		1	0	1	Small checkerboards and tones: spatially congruent versus incongruent (thalamus)
13	16	Bonath	2014	Table 1*A* illusory versus not	20		1	5	6	Small checkerboards and tones: temporal > spatial congruence
	17	Bonath	2014	Table 1*B* synchronous > no illusion		pooled	3	0	3	Small checkerboards and tones: spatial > temporal congruence
14	18	Bushara	2001	Table 1*A* (Fig. 2) AV-Control	12		1	3	4	Tones (100 ms) and colored circles synchrony: detect Auditory then Visual presentation versus Control
	19	Bushara	2001	Table 1*B* (VA-C) five coords		pooled	2	3	5	Tones (100 ms) and colored circles synchrony: detect Visual then Auditory presentation versus Control
	20	Bushara	2001	Table 2*A* interact w/Rt Insula		pooled	2	4	6	Tones and colored circles: correlated functional connections with (and including) the right insula
15	21	Bushara	2003	Table 2*A* collide > pass, strong A-V interact	7		5	3	8	Tone and two visual bars moving: Tone synchrony induce perception they collide (AV interaction) versus pass by
19	28	Calvert	2001	Table 2 superadditive and response depression	10		4	11	15	B/W visual checkerboard reversing and white noise bursts: Synchronous versus not; supradditive and response depression
	29	Calvert	2001	Table 3*A* superadditive only		pooled	6	4	10	B/W visual checkerboard reversing and white noise bursts: Synchronous versus not; supradditive only
	30	Calvert	2001	Table 3*B* response depression only		pooled	3	4	7	B/W visual checkerboard reversing and white noise bursts: Synchronous versus not; response depression only
33	56	Hove	2013	pg 316 AV interaction putamen	14		0	1	1	Interaction between (beep > flash) versus (siren > moving bar); left putamen focus
41	66	Lewis	2000	Table 1	7		2	3	5	Compare speed of tone sweeps to visual dot coherent motion: Bimodal versus unimodal
44	71	Meyer	2007	Table 3 paired A + V versus null	16		3	3	6	Paired screen red flashes with phone ring: paired V (conditioned stimulus) and A (unconditioned) versus null events
	72	Meyer	2007	Table 4 CS+, learned AV association with V-only		pooled	4	6	10	Paired screen flashes with phone ring: View flashes after postconditioned versus null events
53	86	Ogawa	2013a	Table 1 (pg 162 data)	13		1	0	1	AV congruency of pure tone and white dots moving on screen (area left V3A)
69	113	Tanabe	2005	Table 1*A* AV; A then V; not VA	15		10	10	20	Amorphous texture patterns and modulated white noises: Activation during learning delay period (AV)
	114	Tanabe	2005	Table 2A+2B (Fig. 5*a*) AV and VA		pooled	5	6	11	Amorphous texture patterns and modulated white noises: changes after feedback learning (AV and VA)
	115	Tanabe	2005	Table 3A+3B (Fig. 6) AV and VA; delay period		pooled	9	1	10	Amorphous texture patterns and modulated white noises: sustained activity throughout learning (AV and VA)
76	125	Watkins	2006	Figure 4 illusory multisensory interaction	11		0	2	2	Two brief tone pips leads to illusion of two screen flashes (annulus with checkerboard) when only one flash present
	126	Watkins	2006	Table 1 (A enhances V in general)		pooled	5	3	8	Single brief tone pip leads to illusion of single screen flash (annulus with checkerboard) when two flashes present
77	127	Watkins	2007	Figure 3 2 flashes +1 beep illusion	10		0	1	1	Two visual flashes and single audio bleep leads to the illusion of a single flash

Note: Results shown in Figure 3*C* cyan. Refer to Table 1 and text for other details.

**Table 7:** Locations of significant clusters from the meta-analyses involving Congruent and Incongruent audio-visual experimental paradigms (from Tables 5 and 6), indicating major contributing studies to the ALE meta-analysis clusters, weighted centers of mass (*x*, *y*, and *z*) in Talairach coordinates, brain volumes (mm^3^), and ALE values

Condition	Region	Major contributing studies	*x*	*y*	*z*	Volume	ALE value
A. Living audio-visual stimuli single study							
1	L Superior Temporal Sulcus, posterior (pSTS)	8 (6,18,32,40,63,65–67)	−50	−51	10	1448	0.042
2	R Superior Temporal Sulcus	8 (20,31,40,60,65,66,78,79)	48	−37	12	1280	0.035
3	R superior Temporal Gyrus (pSTG)	3 (40,42,45)	55	−19	7	256	0.025
4	L Superior Temporal Gyrus	2 (45,49)	−53	−23	7	144	0.024
B. Nonliving audio-visual stimuli single study							
1	R Anterior Insula	1 (44)	31	19	6	32	0.019
C. Living > Nonliving audio-visual stimuli							
1	L Posterior Superior Temporal Sulcus	2 (40,66)	−50	−52	12	408	2.054
2	R Posterior Superior Temporal Sulcus	1 (66)	51	−35	12	48	1.779
D. Nonliving > Living audio-visual stimuli							
1	R Anterior Insula	1 (44)	31	19	6	32	3.195

Note: (*A*) Single study ALE maps for Living (corrected FWE *P* < 0.05) and (*B*) Nonliving audio-visual interaction sites (corrected FWE *P* < 0.05); plus contrast meta-analyses maps revealing (*C*) Living > Nonliving, and (*D*) Nonliving > Living audio-visual interaction sites (uncorrected *P* < 0.05). The coordinates correspond to foci illustrated in Figure 3*C* (orange and cyan).

A contrast ALE meta-analysis of maps living > nonliving events revealed bilateral pSTS foci as showing significant differential responsiveness (Fig. 3*C*, orange with outline [visible only in left hemisphere model]; Table 7*C*, uncorrected *P* < 0.05). The contrast meta-analysis of nonliving > living congruent audio-visual events revealed the right anterior insula as a common hub of activation (Fig. 2*C*, cyan with white outline; Table 7*D*, uncorrected *P* < 0.05). A main contributing study to this right anterior insula ROI (study #44 Meyer et al. 2007) included screen flashes paired with phone rings as part of a conditioned learning paradigm.

In visual perception literature, a prominent dichotomy of stimulus processing involves “what versus where” streams (Ungerleider et al. 1982; Goodale et al. 1994; Ungerleider and Haxby 1994), which has also been explored in the auditory system (Rauschecker 1998; Kaas and Hackett 1999; Rauschecker and Tian 2000; Clarke et al. 2002; Rauschecker and Scott 2015). A few of the audio-visual interaction studies examined in the present meta-analyses either explicitly or implicitly tested that organization (Sestieri et al. 2006; Plank et al. 2012). However, there were insufficient numbers of studies germane to that dichotomy for conducting a proper meta-analysis along this dimension.

### Vocalization versus Action Event Audio-Visual Interaction Sites

Another stimulus category boundary derived from auditory categorical perception literature was that for processing vocalizations versus action sounds (Fig. 1). To be consistent with that neurobiological model, this category boundary was tested using only living audio-visual sources. This analysis included vocalizations by human or animal sources (Table 8) versus action events (Table 9) including sounds produced by, for example, hand tool use, bodily actions, and persons playing musical instruments. An ALE single study map for experiments using vocalizations revealed four ROIs along the pSTG/pSTS region (Fig. 3*D*, red hues; Table 11*A*, corrected FWE *P* < 0.05). The action event category was initially restricted to using only nonvocalizations (by living things) as auditory stimuli. This initially yielded nine studies that showed audio-visual interaction foci, and no clusters survived the single study ALE meta-analysis map voxel-wise thresholding. Adjusting the study restrictions to include studies that reported using a mix of action events together with some nonliving visual stimuli and some vocalizations as auditory (nonverbal) event stimuli yielded 13 studies (Table 9). A single study ALE map for these action events, which were predominantly nonvocal and depicting living things, revealed one ROI along the left fusiform gyrus (Table 11B, corrected FWE *P* < 0.05).

**Table 8:** Studies included in the Vocalizations category for audio-visual interaction site meta-analyses

Study #	Experiment #				# Subjects	Multiple experiments	Left hem foci	Right hem foci	Number of foci	
40	57	First author	Year	Experimental code and abbreviated task	647		146	117	263	Brief description of experimental paradigm
3	3	Balk	2010	Figure 2 asynchronous versus simultaneous	14		2	1	3	Natural asynchronous versus simultaneous AV speech synchrony (included both contrasts as interaction effects)
5	6	Baumgaertner	2007	Table 3 Action > nonact sentence+video	19		3	0	3	Conjunction spoken sentences (actions>nonactions) AND videos (actions > nonactions)
9	11	Biau	2016	Table 1*A* Interaction; speech synchronous	17		8	0	8	Hand gesture beats versus cartoon disk and speech interaction: synchronous versus asynchronous
11	13	Blank	2013	Figure 2	19		1	0	1	Visual-speech recognition correlated with recognition performance
16	22	Callan	2014	Table 5 AV-Audio (AV10-A10)-(AV6-A6)	16		4	4	8	Multisensory enhancement to visual speech in noise correlated with behavioral results
	23	Callan	2014	Table 6 AV—Visual only		pooled	1	1	2	Multisensory enhancement to visual speech audio-visual versus visual only
17	24	Calvert	1999	Table 1 (Fig. 1)	5		3	4	7	View image of lower face and hear numbers 1 through 10 versus unimodal conditions (AV > Photos, Auditory)
18	25	Calvert	2000	Figure 2 superadd+subadd AVspeech	10		1	0	1	Speech and lower face: supra-additive plus subadditive effects (AV-congruent > A,V > AV-incongruent)
	26	Calvert	2000	Table 1. supradd AVspeech		pooled	4	5	9	Speech and lower face: supra-additive AV enhancement
20	31	Calvert	2003	Table 2A (Fig. 3 blue)	8		13	8	21	Speech and lower face: Moving dynamic speech (phonemes) versus stilled speech frames
22	34	Erickson	2014	Table 1*A* Congruent AV speech	10		2	2	4	McGurk effect (phonemes): congruent AV speech: AV > A and AV > V
	35	Erickson	2014	Table 1*B* McGurk speech		pooled	2	0	2	McGurk speech effect (phonemes)
23	36	Ethofer	2013	Table 1*C* emotion	23		1	2	3	Audiovisual emotional face-voice integration
25	41	Green	2009	Table 4*A* Congruent gesture-speech > gesture or speech	16		1	0	1	Congruent gesture-speech versus gesture with unfamiliar speech and with familiar speech
26	42	Hagan	2013	Table 1 AV emotion, novel over time	18		5	3	8	Affective audio-visual speech: congruent AV emotion versus A, V; unique ROIs over time (MEG)
28	45	Hashimoto	2004	Table 1*G* (Fig. 4*B*, red) Learning Hangul letters to sounds	12		2	1	3	Unfamiliar Hangul letters and nonsense words, learn speech versus tone/noise pairings
29	46	He	2015	Table 3C AV speech foreign (left MTG focus)	20		1	0	1	Intrinsically meaningful gestures with German speech: Gesture-German > Gesture-Russian, German speech only
30	47	He	2018	Table 2 gestures and speech integration	20		1	0	1	Gesture-speech integration: Bimodal speech-gesture versus unimodal gesture with foreign speech and versus unimodal speech
31	50	Hein	2007	Figure 2*C* pSTS, pSTG, mSTG AV-cong	18		0	3	3	Familiar animal images and correct vocalizations (dog: woof-woof)
	52	Hein	2007	Figure 3*B* Foci 2, 3, 4 (blue) artificial/nonliving		pooled	3	0	3	Visual “Fribbles” and backward/underwater distorted animal sounds, learn pairings (blue foci 2,3,4)
	53	Hein	2007	Figure 3*C* congruent living (green)		pooled	3	0	3	Familiar congruent living versus artificial AV object features and animal sounds (green foci 7, 8, 10)
36	59	Jessen	2015	Table 1*A* emotion > neutral AV enhanced	17		1	1	2	Emotional multisensory whole body and voice expressions: AV emotion (anger and fear) > neutral expressions
	60	Jessen	2015	Table 1*D* fear > neutral AV enhanced		pooled	2	1	3	Emotional multisensory whole body and voice expressions: AV fear > neutral expressions
38	62	Kim	2015	Table 2*A* AV > C speech semantic match	15		2	0	2	Moving audio-visual speech perception versus white noise and unopened mouth movements (AV > C)
39	63	Kircher	2009	Figure 3*B*: gesture related activation increase	14		3	1	4	Bimodal gesture-speech versus gesture and versus speech
40	64	Kreifelts	2007	Table 1 voice-face emotion	24		1	2	3	Facial expression and intonated spoken words, judge emotion expressed (AV > A,V; *P* < 0.05 only)
	65	Kreifelts	2007	Table 5 AV increase effective connectivity		pooled	2	4	6	Increased effectiveness connectivity with pSTS and thalamus during AV integration of nonverbal emotional information
42	67	Matchin	2014	Table 1 AV > Aud only (McGurk)	20		2	7	9	McGurk audio-visual speech: AV > A only
	68	Matchin	2014	Table 2 AV > Video only		pooled	9	6	15	McGurk audio-visual speech: AV > V only
45	73	Muller	2012	Supplementary Table 1 effective connectivity changes	27		4	3	7	Emotional facial expression (groaning, laughing) AV integration and gating of information
49	79	Nath	2012	pg 784	14		1	0	1	McGurk effect (phonemes): congruent AV speech correlated with behavioral percept
50	80	Naumer	2008	Figure 2 Table 1*A* max contrast	18		8	6	14	Images of “Fribbles” and learned artificial sounds (underwater animal vocals): post training versus max contrast
	81	Naumer	2008	Figure 3 Table 1*B* pre–post		pooled	5	6	11	Images of “Fribbles” and learned corresponding artificial sounds: Post- versus pretraining session
55	88	Okada	2013	Table 1 AV > A	20		5	4	9	Video of AV > A speech only
56	89	Olson	2002	Table 1*A* synchronized AV > static Vis-only	10		7	4	11	Whole face video and heard words: Synchronized AV versus static V
	90	Olson	2002	Table 1C synchronized AV > desynchronized AV speech		pooled	2	0	2	Whole face video and heard words: Synchronized versus desynchronized
58	93	Raij	2000	Table 1*B* letters and speech sounds	9		2	3	5	Integration of visual letters and corresponding auditory phonetic expressions (MEG study) AV versus (A + V)
60	95	Robins	2008	Table 2 (Fig. 2) AV integration (AV > A and AV > V)	10		2	1	3	Face speaking sentences: angry, fearful, happy, neutral (AV > A,V)
	96	Robins	2008	Table 4*A* (Fig. 5) AV integration and emotion	5		1	4	5	AV faces and spoken sentences expressing fear or neutral valence: AV integration (AV > A,V conditions)
	97	Robins	2008	Table 4*B* emotion effects		pooled	2	0	2	AV faces and spoken sentences expressing fear or neutral valence: Emotional AV-fear > AV-neutral
	98	Robins	2008	Table 4*C* (Fig. 5) fearful AV integration		pooled	1	5	6	AV faces and spoken sentences expressing fear or neutral valence: Fearful-only AV integration
	99	Robins	2008	Table 4*D* AV-only emotion		pooled	1	3	4	AV faces and spoken sentences expressing fear or neutral valence: AV-only emotion
63	104	Sekiyama	2003	Table 3 (fMRI nAV-AV)	8		1	0	1	AV speech, McGurk effect with phonemes (ba, da, ga) and noise modulation: noise-AV > AV (fMRI)
	105	Sekiyama	2003	Table 4 (PET nAV-AV)		pooled	1	3	4	AV speech, McGurk effect with phonemes (ba, da, ga) and noise modulation: noise-AV > AV (PET)
65	109	Stevenson	2009	Table 1*C* (Fig. 8) AVspeech > AVtools	11		1	1	2	Face and speech video: inverse effectiveness (degraded AV speech > AV tool use)
66	110	Straube	2011	Table 3*A* and *B* iconic/metaphoric speech-gestures versus speech, gesture	16		2	2	4	Integration of Iconic and Metaphoric speech-gestures versus speech and gesture
67	111	Straube	2014	p939 Integration foci	16		3	0	3	Integration of iconic hand gesture-speech > unimodal speech and unimodal gesture (healthy control group)
71	118	Van Atteveldt	2004	Table 1*a* letters and speech sounds	16		3	1	4	Familiar letters and their speech sounds: Congruent versus not and Bimodal versus Unimodal
72	119	Van Atteveldt	2007	Table 2A+B (Fig. 2)	12		3	2	5	Single letters and their speech sounds (phonemes): Congruent > Incong; Passive perception, blocked and event-related design
	120	Van Atteveldt	2007	Table 3 (Fig. 2) passive		pooled	1	1	2	Single letters and their speech sounds (phonemes): Congruent > Incong, active perception task
73	122	Van Atteveldt	2010	Table 1B STS; specific adaptation congruent > incong	16		3	1	4	Letter and speech sound pairs (vowels, consonants): Specific adaptation effects
74	123	Van der Wyk	2010	Table 2 AV interaction effects oval/circles +speech/nonspeech	16		3	3	6	Geometric shape modulate with speech (sentences)
75	124	Von Kriegstein	2006	Figure 4*B* after > before voice-face	14		0	4	4	Face and object photos with voice and other sounds: Voice-Face association learning
78	128	Watson	2014a	Table 1*A* AV-adaptation effect (multimodal localizer)	18		0	1	1	Videos of emotional faces and voice: multisensory localizer
	129	Watson	2014a	Table 1*C* AV-adaptation effect, cross-modal adaptation effect		pooled	0	1	1	Videos of emotional faces and voice: crossmodal adaptation effects
79	132	Watson	2014b	Table 4*B* integrative regions (Living and nonliving)	40		0	1	1	Moving objects and videos of faces with corresponding sounds: People-selective integrative region
82	137	Wolf	2014	Table 1 face cartoons + phonemes	16		1	1	2	Drawing of faces with emotional expressions: Supramodal effects with emotional valence

Note: Results shown in Figure 3*D* red hues. Refer to Tables 1 and 2 and text for other details.

**Table 9:** Studies included in the Actions category for audio-visual interaction site meta-analyses

Study #	Experiment #				# Subjects	Multiple experiments	Left hem foci	Right hem foci	Number of foci	
13	19	First author	Year	Experimental code and abbreviated task	205		50	50	100	Brief description of experimental paradigm
6	7	Beauchamp	2004a	Figure 3*J* and *K*, Table 1 first 2 foci only	26		2	0	2	See photographs of tools, animals and hear corresponding sounds versus scrambled images and synthesized rippled sounds
7	8	Beauchamp	2004b	Expt 1 coordinates	8		1	1	2	High-resolution version of 2004a study: AV tool videos versus unimodal (AV > A,V)
8	9	Belardinelli	2004	Table 1 AV semantic congruence	13		6	6	12	Colored images of tools, animals, humans and semantically congruent versus incongruent sounds
27	44	Hasegawa	2004	Table 1*A* (well-trained piano) AV induced by V-only	26		12	6	18	Piano playing: well trained pianists, mapping hand movements to sequences of sound
35	58	James	2011	Table 1*A* bimodal (vs. scrambled)	12		4	2	6	Video of human manual actions (e.g., sawing): Auditory and Visual intact versus scrambled, AV event selectivity
37	61	Jola	2013	Table 1*C* AVcondition dance	12		3	3	6	Viewing unfamiliar dance performance (tells a story by gesture) with versus without music: using intersubject correlation
47	75	Naghavi	2007	Figure 1*C*	23		0	3	3	B/W pictures (animals, tools, instruments, vehicles) and their sounds: Cong versus Incong
57	91	Plank	2012	pg 803 AV congruent effect	15		0	1	1	AV spatially congruent > semantically matching images of natural objects and associated sounds (right STG)
	92	Plank	2012	Table 2*A* spatially congruent-baseline		pooled	5	5	10	Images of natural objects and associated sounds, spatially congruent versus baseline
61	100	Scheef	2009	Table 1 cartoon jump + boing	16		1	2	3	Video of cartoon person jumping and “sonification” of a tone, learn correlated pairings: AV-V and AV-A conjunction
62	101	Schmid	2011	Table 2*E* A effect V (Living and nonliving, pictures)	12		3	4	7	Environmental sounds and matching pictures: reduced activity by A
	102	Schmid	2011	Table 2*F* V competition effect A (reduced activity by a visual object)		pooled	2	2	4	Environmental sounds and matching pictures: reduced activity by V
	103	Schmid	2011	Table 2*G* AV crossmodal interaction × auditory attention		pooled	2	3	5	Environmental sounds and matching pictures: cross-modal interaction and auditory attention
64	106	Sestieri	2006	Table 1 (Fig. 3), AV location match versus semantic	10		2	5	7	B/W images (animal, weapons) and environmental sounds: Match location > recognition
	107	Sestieri	2006	Table 2 AV semantic recognition versus localization		pooled	2	1	3	B/W pictures and environmental sounds: congruent semantic recognition > localization task
65	108	Stevenson	2009	Table 1*B* AVtools > AVspeech	11		1	1	2	Hand tools in use video: inverse effectiveness (degraded AV tool > AV speech)
80	133	Werner	2010	Table 1 superadditive (AV-salience effect)	21		0	3	3	Categorize movies of actions with tools or musical instruments (degraded stimuli); AV interactions both tasks
	134	Werner	2010	Table 2 AV interactions predict behavior		pooled	1	2	3	Categorize movies of actions with tools or musical instruments; AV interactions predicted by behavior
	135	Werner	2010	Table 3*C* superadditive AV due to task		pooled	3	0	3	Categorize movies of actions with tools or musical instruments; Subadditive AV to task

Note: Results shown in Figure 3*D* yellow. Refer to Tables 1 and 2 and text for other details.

The contrast meta-analysis of vocalizations > actions revealed right pSTS and pSTG foci as being preferential for vocalizations (Fig. 3*D*, red with black outlines; Table 11*C*, uncorrected *P* < 0.05). Conversely, the contrast meta-analysis of action > vocalization audio-visual interactions revealed the left fusiform gyrus ROIs (Fig. 3*D*, yellow with black outline; Table 11*D*, uncorrected *P* < 0.05). This left fusiform ROI had a volume of 8 mm^3^, both in the single study and contrast ALE meta-analysis maps. This cluster size fell below some criteria for rigor depending on theoretical interpretation when group differences are diffuse (Tench et al. 2014). Nonetheless, this theoretical processing dissociation existed in at least some single studies, in the single ALE map, and in the contrast ALE map, and was thus at least suggestive of a double-dissociation. A main contributing study to this fusiform ROI (study #62, Schmid et al. 2011) employed a relatively simple task of determining if a colored picture included a match to a presented sound, or vice versa, which involved a wide variety of nonliving but a few living real-world object images. This ROI was consistent in location with the commonly reported fusiform foci involved in functions pertaining to high-level visual object processing (Gauthier et al. 1999; Bar et al. 2001; James and Gauthier 2003).

A subset of the paradigms involving living things and/or vocalizations included emotionally valent stimuli (Table 10). This predominantly including emotional faces with voice (expressing fear, anger, sadness, happiness, and laughter), but also whole body and dance expressions rated for emotional content. These emotionally valent paradigms preferentially activated a portion of the right pSTG (Fig. 3*D*, violet hues; Table 11*E*), when analyzed as a single study ALE map (corrected FWE *P* < 0.05), but also as a contrast meta-analysis with nonemotionally valent paradigms involving living things (mostly control conditions from the same or similar paradigms; data not shown).

**Table 10:** Studies included in the Emotional audio-visual interaction site meta-analyses

Study #	Experiment #				# Subjects	Multiple experiments	Left hem foci	Right hem foci	Number of foci	
9	13	First author	Year	Experimental code and abbreviated task	160		24	29	53	Brief description of experimental paradigm
23	36	Ethofer	2013	Table 1*C* emotion	23		1	2	3	Audiovisual emotional face-voice integration
26	42	Hagan	2013	Table 1 AV emotion, novel over time	18		5	3	8	Affective audio-visual speech: congruent AV emotion versus A, V; unique ROIs over time (MEG)
36	59	Jessen	2015	Table 1*A* emotion > neutral AV enhanced	17		1	1	2	Emotional multisensory whole body and voice expressions: AV emotion (anger and fear) > neutral expressions
	60	Jessen	2015	Table 1*D* fear > neutral AV enhanced		pooled	2	1	3	Emotional multisensory whole body and voice expressions: AV fear > neutral expressions
37	61	Jola	2013	Table 1*C* AVcondition dance	12		3	3	6	Viewing unfamiliar dance performance (tells a story by gesture) with versus without music: using intersubject correlation
40	64	Kreifelts	2007	Table 1 voice-face emotion	24		1	2	3	Facial expression and intonated spoken words, judge emotion expressed (AV > A,V; *P* < 0.05 only)
	65	Kreifelts	2007	Table 5 AV increase effective connectivity		pooled	2	4	6	Increased effectiveness connectivity with pSTS and thalamus during AV integration of nonverbal emotional information
45	73	Muller	2012	Supplementary Table 1 effective connectivity changes	27		4	3	7	Emotional facial expression (groaning, laughing) AV integration and gating of information
60	97	Robins	2008	Table 4*B* emotion effects	5		2	0	2	AV faces and spoken sentences expressing fear or neutral valence: Emotional AV-fear > AV-neutral
	98	Robins	2008	Table 4*C* (Fig. 5) fearful AV integration		pooled	1	5	6	AV faces and spoken sentences expressing fear or neutral valence: Fearful-only AV integration
	99	Robins	2008	Table 4*D* AV-only emotion		pooled	1	3	4	AV faces and spoken sentences expressing fear or neutral valence: AV-only emotion
78	129	Watson	2014a	Table 1*C* AV-adaptation effect, cross-modal adaptation effect	18		0	1	1	Videos of emotional faces and voice: crossmodal adaptation effects
82	137	Wolf	2014	Table 1 face cartoons + phonemes	16		1	1	2	Drawing of faces with emotional expressions: Supramodal effects with emotional valence

Note: Most of these studies used vocalizations as auditory stimuli, and thus was included as a subset of the congruent vocalization category with results shown in Figure 3*D* violet hues. Refer to Tables 1 and 2 and text for other details.

**Table 11:** Locations of significant clusters from the meta-analyses involving Vocalizations and Nonvocal audio-visual experimental paradigms, indicating major contributing studies to the ALE meta-analysis clusters, weighted centers of mass (*x*, *y*, and *z*) in Talairach coordinates, brain volumes (mm^3^), and ALE values

Condition	Region	Major contributing studies	*x*	*y*	*z*	Volume	ALE value
A. Vocal audio-visual stimuli single study							
1	R Superior Temporal Sulcus	19 (20,23,26,31,40,42,45,50,55, 58,60,60,65,66,71,72,74,78,79)	50	−32	11	3040	0.041
2	L Superior Temporal Sulcus (posterior), BA 22	9 (18,22,40,63,66,67,71,73,74)	−54	−47	11	1328	0.034
3	L Superior Temporal Sulcus, BA 41	7 (3,22,31,42,45,50,72)	−49	−21	7	1200	0.035
4	L Superior Temporal Gyrus (posterior), BA 41	4 (45,50,60,65)	−47	−37	11	376	0.030
B. Nonvocal (living) audio-visual stimuli single study							
1	L Fusiform Gyrus (inferior-medial)	1 (62)	−28	−54	−14	8	0.017
C. Vocal > Nonvocal audio-visual stimuli							
	R Posterior Superior Temporal Sulcus	7 (31,40,60,65,66,78,79)	46	−37	13	976	2.530
	R Posterior Superior Temporal Gyrus		54	−26	8	8	1.672
D. Nonvocal > Vocal audio-visual stimuli							
1	L Fusiform Gyrus (inferior-medial)	1 (62)	−28	−54	−14	8	2.400
E. Emotionally valent (mostly vocal) > Nonemotional stimuli							
	R Posterior Superior Temporal Gyrus	3 (26,37,45)	58	−21	8	152	2.391

Note: (*A*) Single study ALE maps for Vocalizations (corrected FWE *P* < 0.05) and (*B*) Action stimuli (corrected FWE *P* < 0.05), plus (*C*) contrast maps revealing interaction sites involving Vocalization > Action and (*D*) Action > Vocalization auditory stimuli (both uncorrected *P* < 0.05). A subset of the Vocal/Living audio-visual stimuli also entailed (*E*) emotionally valent audio-visual stimuli, which was conducted as a single study ALE map (corrected FWE *P* < 0.05). TTG = transverse temporal gyrus (aka HG = Heschl’s gyrus). The coordinates correspond to foci illustrated in Figure 3*D* (red, yellow, and violet hues) (from Tables 8–10).

### Dynamic Visual Motion versus Static Images in Audio-Visual Interactions

We next sought to determine if the use of dynamic visual motion versus static visual images in audio-visual interaction paradigms might reveal differences in processing organizations in the brain. Studies using dynamic-visual stimuli (Table 12), included talking faces, the McGurk effect, hand gestures, bodily gestures, and geometric shapes modulating synchronously with vocals, plus nonvocal drum sounds, musical instruments (e.g., piano), hand tool sounds, tone sweeps, and synthetic tones. Studies using static-visual images (Table 13) involved the matching of pictures of human faces or animals to characteristically associated vocal sounds, plus other forms of photos or drawings (in color, grayscale, or black and white) of faces, animals, objects, or written word/character forms, while excluding stimuli such as flashing screens or light emitting diodes (LEDs). ALE single study maps for experiments utilizing dynamic-visual stimuli (Fig. 3*E*, blue hues; Table 14*A*, corrected FWE *P* < 0.05) and static-visual stimuli (pink hues; Table 14*B*, corrected FWE *P* < 0.05) were constructed. A contrast ALE meta-analysis of dynamic-visual > static-visual revealed significantly greater activation of the right pSTS region (Fig. 3*E*, blue with black outline; Table 14*C*, uncorrected *P* < 0.05). Conversely, the contrast ALE meta-analysis of static-visual > dynamic-visual paradigms preferentially activated the bilateral planum temporale and STG regions (Fig. 3*E*, pink with black outlines; Table 14*D*, uncorrected *P* < 0.05).

**Table 12:** Studies included in the Dynamic-visual stimuli category for audio-visual interaction site meta-analyses

Study #	Experiment #				# Subjects	Multiple experiments	Left hem foci	Right hem foci	Number of foci	
43	62	First author	Year	Experimental code and abbreviated task	682		177	148	325	Brief description of experimental paradigm
2	2	Alink	2008	Table 1*c* spheres move to drum sounds	10		4	6	10	Visual spheres and drum sounds moving: crossmodal dynamic capture versus conflicting motion
3	3	Balk	2010	Figure 2 asynchronous versus simultaneous	14		2	1	3	Natural asynchronous versus simultaneous AV speech synchrony (included both contrasts as interaction effects)
4	4	Baumann	2007	Table 1*B* coherent V + A versus A	12		2	1	3	Visual dots 16% coherent motion and in-phase acoustic noise > stationary acoustic sound
	5	Baumann	2007	Table 2*B*		pooled	15	12	27	Moving acoustic noise and visual dots 16% in-phase coherent > random dot motion
5	6	Baumgaertner	2007	Table 3 Action > nonact sentence+video	19		3	0	3	Conjunction spoken sentences (actions > nonactions) AND videos (actions > nonactions)
7	8	Beauchamp	2004b	Expt 1 coordinates	8		1	1	2	High-resolution version of 2004a study: AV tool videos versus unimodal (AV > A,V)
9	11	Biau	2016	Table 1*A* Interaction; speech synchronous	17		8	0	8	Hand gesture beats versus cartoon disk and speech interaction: synchronous versus asynchronous
11	13	Blank	2013	Figure 2	19		1	0	1	Visual-speech recognition correlated with recognition performance
15	21	Bushara	2003	Table 2*A* collide > pass, strong A-V interact	7		5	3	8	Tone and two visual bars moving: Tone synchrony induce perception they collide (AV interaction) versus pass by
16	22	Callan	2014	Table 5 AV-Audio (AV10-A10)-(AV6-A6)	16		4	4	8	Multisensory enhancement to visual speech in noise correlated with behavioral results
	23	Callan	2014	Table 6 AV—Visual only		pooled	1	1	2	Multisensory enhancement to visual speech audio-visual versus visual only
18	25	Calvert	2000	Figure 2 superadd+subadd AVspeech	10		1	0	1	Speech and lower face: supra-additive plus subadditive effects (AV-congruent > A,V > AV-incongruent)
	26	Calvert	2000	Table 1. supradd AVspeech		pooled	4	5	9	Speech and lower face: supra-additive AV enhancement
20	31	Calvert	2003	Table 2*A* (Fig. 3 blue)	8		13	8	21	Speech and lower face: Moving dynamic speech (phonemes) versus stilled speech frames
21	32	DeHaas	2013	Table 1*A* AVcong—Visual	15		3	3	6	Video clips of natural scenes (animals, humans): AV congruent versus Visual
22	34	Erickson	2014	Table 1*A* Congruent AV speech	10		2	2	4	McGurk effect (phonemes): congruent AV speech: AV > A and AV > V
		Erickson	2014	Table 1*B* McGurk speech		pooled	2	0	2	McGurk speech effect (phonemes)
23	36	Ethofer	2013	Table 1*C* emotion	23		1	2	3	Audiovisual emotional face-voice integration
25	41	Green	2009	Table 4*A* Congruent gesture-speech > gesture or speech	16		1	0	1	Congruent gesture-speech versus gesture with unfamiliar speech and with familiar speech
26	42	Hagan	2013	Table 1 AV emotion, novel over time	18		5	3	8	Affective audio-visual speech: congruent AV emotion versus A, V; unique ROIs over time (MEG)
27	44	Hasegawa	2004	Table 1*A* (well trained piano) AV induced by V-only	26		12	6	18	Piano playing: well trained pianists, mapping hand movements to sequences of sound
29	46	He	2015	Table 3*C* AV speech foreign (left MTG focus)	20		1	0	1	Intrinsically meaningful gestures with German speech: Gesture-German > Gesture-Russian, German speech only
30	47	He	2018	Table 2, GSI, left MTG, gestures and speech integration	20		1	0	1	Gesture-speech integration: Bimodal speech-gesture versus unimodal gesture with foreign speech and versus unimodal speech
35	58	James	2011	Table 1*A* bimodal (vs scrambled)	12		4	2	6	Video of human manual actions (e.g., sawing): Auditory and Visual intact versus scrambled, AV event selectivity
36	59	Jessen	2015	Table 1*A* emotion > neutral AV enhanced	17		1	1	2	Emotional multisensory whole body and voice expressions: AV emotion (anger and fear) > neutral expressions
	60	Jessen	2015	Table 1*D* fear > neutral AV enhanced		pooled	2	1	3	Emotional multisensory whole body and voice expressions: AV fear > neutral expressions
37	61	Jola	2013	Table 1*C* AVcondition dance	12		3	3	6	Viewing unfamiliar dance performance (tells a story by gesture) with versus without music: using intersubject correlation
38	62	Kim	2015	Table 2*A* AV > C speech semantic match	15		2	0	2	Moving audio-visual speech perception versus white noise and unopened mouth movements
39	63	Kircher	2009	Figure 3*B*: gesture-related activation increase	14		3	1	4	Bimodal gesture-speech versus gesture and versus speech
40	64	Kreifelts	2007	Table 1 voice-face emotion	24		1	2	3	Facial expression and intonated spoken words, judge emotion expressed (AV > A,V; *P* < 0.05 only)
	65	Kreifelts	2007	Table 5 AV increase effective connectivity		pooled	2	4	6	Increased effectiveness connectivity with pSTS and thalamus during AV integration of nonverbal emotional information
41	66	Lewis	2000	Table 1	7		2	3	5	Compare speed of tone sweeps to visual dot coherent motion: Bimodal versus unimodal
42	67	Matchin	2014	Table 1 AV > Aud only (McGurk)	20		2	7	9	McGurk audio-visual speech: AV > A only
	68	Matchin	2014	Table 2 AV > Video only		pooled	9	6	15	McGurk audio-visual speech: AV > V only
43	70	McNamara	2008	Table (BA44 and IPL)	12		2	2	4	Videos of meaningless hand gestures and synthetic tone sounds: Increases in functional connectivity with learning
49	79	Nath	2012	pg 784	14		1	0	1	McGurk effect (phonemes): congruent AV speech correlated with behavioral percept
54	87	Ogawa	2013b	Table 1 3D > 2D and surround > monaural effects	16		3	4	7	Cinematic 3D > 2D video and surround sound > monaural while watching a movie (“The Three Musketeers”)
55	88	Okada	2013	Table 1 AV > A	20		5	4	9	Video of AV > A speech only
56	89	Olson	2002	Table 1*A* synchronized AV > static Vis-only	10		7	4	11	Whole face video and heard words: Synchronized AV versus static V
	90	Olson	2002	Table 1*C* synchronized AV > desynchronized AV speech		pooled	2	0	2	Whole face video and heard words: Synchronized versus desynchronized
59	94	Regenbogen	2017	Table 2*A* degraded > clear Multisensory versus unimodal input	29		5	6	11	Degraded > clear AV versus both visual and auditory unimodal visual real-world object-in-action recognition
60	95	Robins	2008	Table 2 (Fig. 2) AV integration (AV > A and AV > V)	10		2	1	3	Face speaking sentences: angry, fearful, happy, neutral (AV > A,V)
	96	Robins	2008	Table 4*A* (Fig. 5) AV integration and emotion		pooled	1	4	5	AV faces and spoken sentences expressing fear or neutral valence: AV integration (AV > A,V conditions)
	97	Robins	2008	Table 4*B* emotion effects		pooled	2	0	2	AV faces and spoken sentences expressing fear or neutral valence: Emotional AV-fear > AV-neutral
	98	Robins	2008	Table 4*C* (Fig. 5) fearful AV integration		pooled	1	5	6	AV faces and spoken sentences expressing fear or neutral valence: Fearful-only AV integration
	99	Robins	2008	Table 4*D* AV-only emotion		pooled	1	3	4	AV faces and spoken sentences expressing fear or neutral valence: AV-only emotion
61	100	Scheef	2009	Table 1 cartoon jump + boing	16		1	2	3	Video of cartoon person jumping and “sonification” of a tone, learn correlated pairings: AV-V and AV-A conjunction
63	104	Sekiyama	2003	Table 3 (fMRI nAV-AV)	8		1	0	1	AV speech, McGurk effect with phonemes (ba, da, ga) and noise modulation: noise-AV > AV (fMRI)
	105	Sekiyama	2003	Table 4 (PET nAV-AV)		pooled	1	3	4	AV speech, McGurk effect with phonemes (ba, da, ga) and noise modulation: noise-AV > AV (PET)
65	108	Stevenson	2009	Table 1*B* 2 AVtools > AVspeech	11		1	1	2	Hand tools in use video: inverse effectiveness (degraded AV tool > AV speech)
	109	Stevenson	2009	Table 1*C* (Fig. 8) AVspeech > AVtools		pooled	1	1	2	Face and speech video: inverse effectiveness (degraded AV speech > AV tool use)
66	110	Straube	2011	Table 3*A* and *B* iconic/metaphoric speech-gestures versus speech, gesture	16		2	2	4	Integration of Iconic and Metaphoric speech-gestures versus speech and gesture
67	111	Straube	2014	p939 Integration foci	16		3	0	3	Integration of iconic hand gesture-speech > unimodal speech and unimodal gesture (healthy control group)
74	123	Van der Wyk	2010	Table 2 AV interaction effects oval/circles+speech/nonspeech	16		3	3	6	Geometric shape modulate with speech (sentences)
78	128	Watson	2014a	Table 1*A* AV-adaptation effect (multimodal localizer)	18		0	1	1	Videos of emotional faces and voice: multisensory localizer
	129	Watson	2014a	Table 1*C* AV-adaptation effect, cross-modal adaptation effect		pooled	0	1	1	Videos of emotional faces and voice: crossmodal adaptation effects
79	130	Watson	2014b	Table 1 AV > baseline (Living and nonliving)	40		3	5	8	Moving objects and videos of faces with corresponding sounds: AV > baseline
	131	Watson	2014b	Table 4*A* integrative regions (Living and nonliving)		pooled	2	2	4	Moving objects and videos of faces with corresponding sounds: Integrative regions (AV > A,V)
	132	Watson	2014b	Table 4*B* integrative regions (Living and nonliving)		pooled	0	1	1	Moving objects and videos of faces with corresponding sounds: People-selective integrative region
80	133	Werner	2010	Table 1 superadditive (AV-salience effect)	21		0	3	3	Categorize movies of actions with tools or musical instruments (degraded stimuli); AV interactions both tasks
	134	Werner	2010	Table 2 AV interactions predict behavior		pooled	1	2	3	Categorize movies of actions with tools or musical instruments; AV interactions predicted by behavior
	135	Werner	2010	Table 3*C* superadditive AV due to task		pooled	3	0	3	Categorize movies of actions with tools or musical instruments; Subadditive AV to task

Note: Results shown in Figure 3*E* blue. Refer to Tables 1 and 2 and text for other details.

**Table 13:** Studies included in the Static-visual stimuli category for audio-visual interaction site meta-analyses

Study #	Experiment #				# Subjects	Multiple experiments	Left hem foci	Right hem foci	Number of foci	
26	39	First author	Year	Experimental code and abbreviated task	405		106	89	195	Brief description of experimental paradigm
1	1	Adams	2002	Expt 1 Table 3 A + V (aud coords only)	12		5	1	6	A and V commonly showing subordinate > basic object name verification (words with pictures or environmental sounds)
6	7	Beauchamp	2004a	Figure 3*J* and *K*, Table 1 first 2 foci only	26		2	0	2	See photographs of tools, animals and hear corresponding sounds versus scrambled images and synthesized rippled sounds
8	9	Belardinelli	2004	Table 1 AV semantic congruence	13		6	6	12	Colored images of tools, animals, humans and semantically congruent versus incongruent sounds
17	24	Calvert	1999	Table 1 (Fig. 1)	5		3	4	7	View image of lower face and hear numbers 1 through 10 versus unimodal conditions (AV > Photos, Auditory)
24	37	Gonzalo	2000	Table 1 AV > AVincon music and Chinese ideograms	14		1	1	2	Learn novel Kanji characters and musical chords, activity increases over time for consistent AV pairings
	39	Gonzalo	2000	Table 3 AV consistent versus Aud		pooled	1	1	2	Learn novel Kanji characters and musical chords, learn consistent (vs inconsistent) pairings versus auditory only
28	45	Hashimoto	2004	Table 1*G* (Fig. 4*B*, red) Learning Hangul letters to sounds	12		2	1	3	Unfamiliar Hangul letters and nonsense words, learn speech versus tone/noise pairings
31	49	Hein	2007	Figure 2*B* AV-artificial/nonliving	18		0	1	1	B/W images of artificial objects (“fribbles”) and animal vocalizations versus unimodal A, V
	50	Hein	2007	Figure 2*C* pSTS, pSTG, mSTG AV-cong		pooled	0	3	3	Familiar animal images and correct vocalizations (dog: woof-woof)
	52	Hein	2007	Figure 3*B* Foci 2, 3, 4 (blue) artificial/nonliving		pooled	3	0	3	Visual “Fribbles” and backward/underwater distorted animal sounds, learn pairings (blue foci 2,3,4)
	53	Hein	2007	Figure 3*C* congruent living (green)		pooled	3	0	3	Familiar congruent living versus artificial AV object features and animal sounds (green foci 7, 8, 10)
32	54	Hocking	2008	pg 2444 verbal	18		2	0	2	(pSTS mask) Color photos, written names, auditory names, environmental sounds conceptually matched “amodal”
34	57	James	2003	Figure 2	12		0	1	1	Activation by visual objects (“Greebles”) associated with auditory features (e.g., buzzes, screeches); (STG)
45	73	Muller	2012	Table S1 effective connectivity changes	27		4	3	7	Emotional facial expression (groaning, laughing) AV integration and gating of information
47	75	Naghavi	2007	Figure 1*C*	23		0	3	3	B/W pictures (animals, tools, instruments, vehicles) and their sounds: Cong versus Incong
48	76	Naghavi	2011	Figure 2*A* cong = incon	30		1	0	1	B/W drawings of objects (living and non) and natural sounds (barking, piano): congruent = incongruent encoding
	77	Naghavi	2011	Figure 2*B* congruent > incongruent		pooled	0	1	1	B/W drawings of objects (living and non) and natural sounds (barking, piano): congruent > incongruent encoding
50	80	Naumer	2008	Figure 2 Table 1*A* max contrast	18		8	6	14	Images of “Fribbles” and learned artificial sounds (underwater animal vocals): post training versus max contrast
	81	Naumer	2008	Figure 3 Table 1*B* pre–post		pooled	5	6	11	Images of “Fribbles” and learned corresponding artificial sounds: Post- versus Pretraining session
51	83	Naumer	2011	Figure 3*C*	10		1	0	1	Photographs of objects (living and non) and related natural sounds
52	85	Noppeny	2008	Table 3 AV congruent sounds/words	17		4	0	4	Speech sound recognition through AV priming, environmental sounds and spoken words: Congruent > incongruent
57	91	Plank	2012	pg 803 AV congruent effect	15		0	1	1	AV spatially congruent > semantically matching images of natural objects and associated sounds (right STG)
	92	Plank	2012	Table 2*A* spatially congruent-baseline		pooled	5	5	10	Images of natural objects and associated sounds, spatially congruent versus baseline
58	93	Raij	2000	Table 1*B* letters and speech sounds	9		2	3	5	Integration of visual letters and corresponding auditory phonetic expressions (MEG study) AV versus (A + V)
62	101	Schmid	2011	Table 2*E* A effect V (Living and nonliving, pictures)	12		3	4	7	Environmental sounds and matching pictures: reduced activity by A
	102	Schmid	2011	Table 2*F* V competition effect A (reduced activity by a visual object)		pooled	2	2	4	Environmental sounds and matching pictures: reduced activity by V
	103	Schmid	2011	Table 2*G* AV crossmodal interaction × auditory attention		pooled	2	3	5	Environmental sounds and matching pictures: cross-modal interaction and auditory attention
64	106	Sestieri	2006	Table 1 (Fig. 3), AV location match versus semantic	10		2	5	7	B/W images (animal, weapons) and environmental sounds: Match location > recognition
	107	Sestieri	2006	Table 2 AV semantic recognition versus localization		pooled	2	1	3	B/W pictures and environmental sounds: congruent semantic recognition > localization task
69	113	Tanabe	2005	Table 1*A* AV; A then V; not VA	15		10	10	20	Amorphous texture patterns and modulated white noises: Activation during learning delay period (AV)
	114	Tanabe	2005	Table 2A+2B (Fig. 5*a*) AV and VA		pooled	5	6	11	Amorphous texture patterns and modulated white noises: changes after feedback learning (AV and VA)
	115	Tanabe	2005	Table 3A+3B (Fig. 6) AV and VA; delay period		pooled	9	1	10	Amorphous texture patterns and modulated white noises: sustained activity throughout learning (AV and VA)
70	117	Taylor	2006	Figure 1*A* and *B*, Figure 1*C* and *D* (living > nonliving)	15		2	0	2	Color photos (V), environmental sounds and spoken words (A): Cong AV versus Incong (living objects)
71	118	Van Atteveldt	2004	Table 1*a* letters and speech sounds	16		3	1	4	Familiar letters and their speech sounds: Congruent versus not and Bimodal versus Unimodal
72	119	Van Atteveldt	2007	Table 2A+B (Fig. 2)	12		3	2	5	Single letters and their speech sounds (phonemes): Congruent > Incong; Passive perception, blocked and event-related design
	120	Van Atteveldt	2007	Table 3 (Fig. 2) passive		pooled	1	1	2	Single letters and their speech sounds (phonemes): Congruent > Incongruent, active perception task
73	122	Van Atteveldt	2010	Table 1*B* STS; specific adaptation congruent > incong	16		3	1	4	Letter and speech sound pairs (vowels, consonants): Specific adaptation effects
75	124	Von Kriegstein	2006	Figure 4B after > before voice-face	14		0	4	4	Face and object photos with voice and other sounds: Voice-Face association learning
82	137	Wolf	2014	Table 1 face cartoons + phonemes	16		1	1	2	Drawing of faces with emotional expressions: Supramodal effects with emotional valence

Note: Results shown in Figure 3*E* pink. Refer to Tables 1 and 2 and text for other details.

**Table 14:** Locations of significant clusters from the meta-analyses involving Dynamic-visual and Static-visual audio-visual experimental paradigms, indicating major contributing studies to the ALE meta-analysis clusters, weighted centers of mass (*x*, *y*, and *z*) in Talairach coordinates, brain volumes (mm^3^), and ALE values

Condition	Region	Major contributing studies	*x*	*y*	*z*	Volume	ALE value
A. Dynamic-visual audio-visual stimuli single study							
1	R Posterior Superior Temporal Sulcus	9 (20,40,60,60,65,66,74,78,79)	48	−36	11	1312	0.037
2	L Posterior Superior Temporal Sulcus	6 (18,40,65,66,67,79)	−51	−49	10	928	0.035
3	L Posterior Superior Temporal Gyrus	2 (22,74)	−58	−38	12	136	0.027
4	R Superior Temporal Gyrus		58	−17	8	32	0.024
B. Static-visual audio-visual stimuli single study							
1	L Transverse Temporal Gyrus/Planum Temporale	5 (31,45,50,57,72)	−47	−22	7	552	0.031
2	R Superior Temporal Gyrus/Planum Temporale	2 (45,72)	53	−20	8	288	0.023
3	R Superior Temporal Gyrus		58	−29	11	120	0.021
C. Dynamic-visual > Static-visual audio-visual stimuli							
1	R Superior Temporal Gyrus/Sulcus	4 (40,60,60,66)	46	−37	12	392	2.287
D. Static-visual > Dynamic-visual audio-visual stimuli							
1	L Superior Temporal Gyrus/Planum Temporale/TTG	4 (31,50,57,72)	−46	−22	7	480	2.620
2	R Superior Temporal Gyrus (posterior)		58	−28	11	128	2.308
3	R Superior Temporal Gyrus/Sulcus		52	−20	3	64	2.254
4	R Transverse Temporal Gyrus		50	−18	8	24	1.739
5	R Transverse Temporal Gyrus		52	−18	12	8	1.863

Note: Single study ALE maps for (*A*) Dynamic-visual stimuli (corrected FWE *P* < 0.05) and (*B*) Static-visual stimuli (nonmoving images) (corrected FWE *P* < 0.05), plus (*C*) contrast maps of interaction sites revealing Dynamic-visual > Static-visual, and (*D*) Static-visual > Dynamic-visual audio-visual stimuli (both uncorrected *P* < 0.05). The coordinates correspond to foci illustrated in Figure 3*E* (blue and pink hues) (from Tables 12 and 13).

Analyses of the dynamic-visual versus static-visual were further conducted separately for those experimental paradigms using artificial versus natural stimuli. With the exception of natural dynamic-visual studies (*n* = 37 of the 43 in Table 12), the other subcategories had too few studies for the recommended 17–20 study minimum for meta-analysis. Nonetheless, the artificial static-visual (*n* = 12) meta-analysis revealed clusters that overlapped with the outcomes using the respective full complement of studies, while the natural dynamic-visual (*n* = 37) meta-analysis similarly revealed clusters that overlapped with the respective full complement of studies. Thus, audio-visual events involving dynamic visual motion (and mostly natural stimuli) generally recruited association cortices situated roughly between auditory and visual cortices, while audio-visual interactions involving static (iconic) visual images (and mostly artificial stimuli) generally recruited regions located closer to auditory cortex proper along the pSTG and planum temporale bilaterally.

## Discussion

The present meta-analyses examined a wide variety of published human neuroimaging studies that revealed some form of audio-visual “interaction” in the brain, entailing responses beyond or different from the corresponding unisensory auditory and/or visual stimuli alone. One objective was to test several tenets regarding potential brain organizations or architectures that may develop for processing different categories of audio-visual event types at a semantic level. The tenets were borne out of recent ethologically derived unisensory hearing perception literature (Brefczynski-Lewis and Lewis 2017). This included a taxonomic model of semantic categories of natural sound-producing events (i.e., Fig. 1), but here being applied to testing specific hypotheses in the realm of multisensory (audio-visual) processing. The category constructs were derived with the idea of identifying putative cortical “hubs” that could be further applied to, and tested by, various neurocomputational models of semantic knowledge and multisensory processing.

Providing modest support for our first hypothesis, contrast ALE meta-analyses revealed a double-dissociation of brain regions preferential for the processing of living versus nonliving (mostly artificial sources) audio-visual interaction events at a category level (Fig. 3*C*, orange vs. cyan). These results implicated the bilateral pSTS complexes versus the right anterior insula as processing hubs, respectively, which are further addressed below in Embodied Representations of Audio-Visual Events. Providing modest support for our second hypothesis, contrast ALE meta-analyses revealed a double-dissociation of brain regions preferential for the processing of audio-visual interaction events involving vocalizations versus actions, respectively (Fig. 3*D*, red vs. yellow). These results implicated the bilateral planum temporale, pSTG, and pSTS complexes versus the left fusiform cortex, respectively, which is also further addressed in Embodied Representations of Audio-Visual Events below.

Providing strong support for our third hypothesis, different cortices were preferential for processing audio-visual interactions that involved dynamic-visual (video) versus static-visual (iconic images) as visual stimuli (Fig. 3*E*, blue vs. pink). This finding is addressed further below in the context of parallel multisensory processing hierarchies in the section ``Dynamic-Visual versus Static-Visual Images and Audio-Visual Interaction Processing''. The original volumes of the ROIs identified herein (comprising clusters in Tables 4,7,11, and14, and depicted in Fig. 3) are available in Supplementary Material. These ROI volumes should facilitate the generation and testing of new hypotheses, especially as they pertain to neurocomputational theories of semantic knowledge representation, which is a topic addressed in below in the section ``*Semantic processing and neurocomputational models of cognition*''. This is followed by a discussion that considers various limitations of the present meta-analysis studies.

Upon inspection of Figure 3*C*–*E*, only ventral cortices, as opposed to dorsal cortices (e.g., superior to the lateral sulcus), revealed activation foci that were preferential for any of the different semantic categories of audio-visual events. In particular, neither the bilateral IFC foci for congruent versus incongruent audio-visual interactions (Fig. 3*B*, black/white), nor the frontal or parietal cortices (Fig. 3*A*, purple), revealed any differential activation along the semantic category dimensions tested. This was generally consistent with the classic ventral “what is it” (perceptual identification of objects) versus dorsal “where is it” (sensory transformations for guided actions directed at objects) dichotomy observed in both vision and auditory neuroimaging and primate literature (Goodale and Milner 1992; Ungerleider and Haxby 1994; Belin and Zatorre 2000; Sestieri et al. 2006). While dorsal cortical regions such a bilateral parietal cortices and noncortical regions such as the cerebellum were reported to be revealing audio-visual interaction effects by many studies, their involvement appeared to relate more to task demands, spatial processing, and task difficulty rather than semantic category of the audio-visual events per se. Dorsal cortical networks are also implicated in various components of attention. While some form of sensory attention was involved in nearly all of the experimental paradigms, the specific effects of different types or degrees of sensory attention was not a measurable dimension across the studies, and thus fell outside the scope of the present study.

###  

#### Embodied Representations of Audio-Visual Events

One of the tenets regarding the taxonomic category model of real-world hearing perception was that “natural sounds are embodied when possible” (Brefczynski-Lewis and Lewis 2017), and this tenet appears to also apply to the context of cortical organizations for processing audio-visual interactions at a semantic category level. This is further addressed below by region in the context of the pSTS complexes for embodiable representations, and of the right anterior insula focus for nonembodiable nonliving and artificial audio-visual event perception.

##### The pSTS complexes and audio-visual motion processing

The bilateral pSTS complexes were significantly more involved with processing audio-visual interactions associated with events by living things, by stimuli involving vocalizations, and by dynamic-visual (vs static-visual image) audio-visual events (cf. Fig. 3*C*–*E*, orange, red, and blue). Although these respective foci were derived by several overlapping studies, the meta-analysis results support the notion that the lateral temporal cortices are the primary loci for complex natural motion processing (Calvert et al. 2000; Beauchamp, Argall, et al. 2004; Beauchamp, Lee, et al. 2004; Calvert and Lewis 2004; Lewis et al. 2004; Taylor et al. 2006, 2009; Martin 2007): More specifically, the pSTS complexes are thought to play a prominent perceptual role in transforming the spatially and temporally dynamic features of natural auditory and visual action information together into a common neural code, which may then facilitate cross-modal interactions and integration from a “bottom-up” intermodal invariant sensory perspective. An earlier image-based (as opposed to coordinate-based) meta-analysis using a subset of these paradigms (Lewis 2010) further highlighted the idea that the pSTS complexes may form a temporal reference frame for probabilistically comparing the predicted or expected incoming auditory and/or visual information based on what actions have already occurred.

From a “top-down” cognitive perspective, however, words and phrases that depict human actions, and even imagining complex audio and/or visual actions, are reported to lead to activation of the pSTS regions (Kellenbach et al. 2003; Tettamanti et al. 2005; Kiefer et al. 2008; Noppeney et al. 2008). Furthermore, the pSTS complexes are known to be recruited by a variety of sensory-perceptual tasks in congenitally blind and in congenitally deaf individuals (Burton et al. 2004; Pietrini et al. 2004; Amedi et al. 2007; Patterson et al. 2007; Capek et al. 2008, 2010; Lewis, Frum, et al. 2011), suggesting that aspects of their basic functional roles are not dependent on bimodal sensory input outright. To reconcile these findings, one hypothesis was that some cortical regions may develop to perform amodal or metamodal operations (Pascual-Leone and Hamilton 2001). More specifically, different patches of cortex, such as the pSTS, may innately develop to contain circuitry predisposed to compete for the ability to perform particular types of operations or computations useful to the observer regardless of the modality (or presence) of sensory input. Thus, the organization of the multisensory brain may be influenced as much, if not more, by internal processing factors than by specific external sensory experiences per se. This interpretation reflects another tenet regarding the taxonomic category model of real-world hearing perception that “metamodal operators guide sound processing network organizations” (Brefczynski-Lewis and Lewis 2017), but here applying to the processing of audio-visual interactions at a semantic category level.

Another interpretation regarding the functions of the bilateral pSTS complexes is that they are more heavily recruited by living and dynamic audio-visual events simply because of their greater life-long familiarity in adult observers. They may reflect an individual’s experiences and habits of extracting subtle nuances from day-to-day real-world interactions, including other orally communicating people as prevalent sources of multisensory events. Ostensibly, this experiential multisensory process would start from the time of birth when there becomes a critical need to interact with human caretakers. Consistent with this interpretation is that the pSTS complexes have prominent roles in social cognition, wherein reading subtleties of human expressions and body language is often highly relevant for conveying information that guides effective social interactions (Pelphrey et al. 2004; Jellema and Perrett 2006; Zilbovicius et al. 2006).

Embodied cognition models (also called grounded cognition) posit that perception of natural events (social or otherwise) is at least in part dependent on modal simulations, bodily states, and situated actions (Barsalou 2008). The discovery of mirror neuron systems (MNS) and echo-mirror neuron (ENS) systems (Rizzolatti and Arbib 1998; Rizzolatti and Craighero 2004; Molenberghs et al. 2012) have been recognized as having major implications for explaining many cognitive functions, including action understanding, imitation and empathy. Such neuronal systems, which often include the bilateral pSTS complexes, are proposed to mediate elements of the perception of sensory events as they relate to one’s own repertoire of dynamic visual action-producing and sound-producing motoric events (Gazzola et al. 2006; Lahav et al. 2007; Galati et al. 2008; Engel et al. 2009; Lewis et al. 2018). Thus, the pSTS complexes may reflect metamodal cortices that typically develop to process natural multisensory events, which especially include dynamic actions by living things (including vocalizations) that are interpreted for meaningfulness (and possibly intent) based on embodiment strategies by the brain. Notwithstanding, the dynamic viewable motions and sounds produced by nonliving things and artificial stimulus events are arguably less embodiable or mimicable than those by living things. The pSTG/pSTS complexes were not preferentially activated by nonliving and artificial multisensory events. Rather, this event category preferentially recruited the right anterior insula, as addressed next.

##### The right anterior insula and nonliving/artificial audio-visual interaction processing

The right anterior insula emerged as a cortical hub that was preferentially involved in processing nonliving and largely artificial audio-visual sources, which are typically deemed as being nonembodiable. Moreover, unlike the pSTS complexes, the right anterior insula did not show significant sensitivity to the dynamic-visual versus static-visual image stimulus dimension, suggesting that intermodal invariant cues were not a major driving factor in its recruitment. Interestingly, the mirror opposite left anterior insula showed preferential activation for incongruent versus congruent audio-visual stimuli (cf. Fig. 3*B*,*C*).

On a technical note, portions of the claustrum are located very close to the anterior insulae, and activation of the claustrum may have contributed to the anterior insula foci in several neuroimaging studies, and thus also in this meta-analysis. The enigmatic claustrum is reported to have a role in integrative processes that require the analysis of the “content” of the stimuli, and in coordinating the rapid integration of object attributes across different modalities that lead to coherent conscious percepts (Crick and Koch 2005; Naghavi et al. 2007).

Embodiment encoding functions have been ascribed to the anterior insula in representing “self” versus “nonself.” For instance, the anterior insulae, which receive input from numerous cortical areas, have reported roles in multimodal integrative functions, rerepresentation of interoceptive awareness of bodily states, cognitive functions, and metacognitive functions (Craig 2009, 2010; Menon and Uddin 2010), and in social emotions that may function to help establish “other-related” states (Singer et al. 2004; Lamm and Singer 2010). The right lateralized anterior insula activation has further been reported to be recruited during nonverbal empathy-related processing such as with compassion meditation, which places an emphasis on dissolving the “self-versus-other” boundary (Lutz et al. 2008). Moreover, dysfunction of the anterior insulae has been correlated with an inability to differentiate the self from the nonself in patients with schizophrenia (Cascella et al. 2011; Shura et al. 2014).

Although the anterior insula territories are commonly associated with affective states, visceral responses, and the processing of feelings (Damasio 2001; Critchley et al. 2004; Dalgleish 2004; Mutschler et al. 2009; Cacioppo 2013), the emotionally valent paradigms in this meta-analysis did not yield significant differential audio-visual interaction effects in the right (or left) insula, but rather only along the right pSTG. Though speculative, the anterior insula(e) may be subserving the mapping of events that are heightened by relatively “nonembodiable” multisensory events (notably nonliving and artificial sources) with differential activation depending on the perceived relatedness to self. This outcome will likely be a topic of interest for future studies, including neurocomputational modeling of cognition, which is addressed in a later section after first considering parallel multisensory processing hierarchies.

#### Dynamic-Visual versus Static-Visual Images and Audio-Visual Interaction Processing

The double-dissociation of cortical hubs for processing dynamic-visual versus static-visual audio-visual interactions was consistent with notion of parallel processing hierarchies. The experimental paradigms using video typically included dynamic intermodal invariant cross-modal cues (mostly by living things), where the audio and visual stimuli were either perceived to be coming from roughly the same region of space, moving along similar spatial trajectories, and/or had common temporal synchrony and modulations in stimulus intensity or change. These correlated physical changes in photic and acoustic energy attributes are likely to serve to naturally bind audio-visual interactions, consistent with bottom-up Hebbian-like learning mechanisms. Such stimuli preferentially recruited circuitry of the bilateral pSTS complexes (Fig. 3*E*, blue vs. pink), as addressed earlier.

In direct contrast to dynamic-visual stimuli, static-visual images (e.g., pictures, characters, and drawings) can have symbolic congruence with sound that must be learned to be associated with, and having few or no cross-modal invariant cues, thereby placing greater emphasis on declarative memory and related semantic-level matching mechanisms. The dynamic versus static visual stimulus dimension was further assessed using a subset of natural-only versus artificial stimuli. While there were insufficient numbers of studies in three of the subgroups for definitive meta-analysis results (data not shown), the outcomes suggested a bias for the dynamic-visual stimuli clusters being driven by natural stimuli while the static-visual stimuli clusters may have been driven more by images involving relatively artificial stimuli (e.g., checkerboards, dots, circles, texture patterns). Regardless, a double-dissociation was evident.

Another consideration regarding the dynamic/natural versus static/artificial processing was depth-of-encoding. The greater depth required for encoding for subordinate versus basic level information is reported to recruit greater expanses of cortices along the anterior temporal lobes (Adams and Janata 2002; Tranel et al. 2003; Tyler et al. 2004). For instance, associating a picture of an iconic dog to the sound “woof” represents a “basic” level of semantic matching, while matching the specific and more highly familiar image of one’s pet Tibetan terrier to her particular bark to be let outside would represent a “subordinate” level of matching that is regarded as having greater depth in its encoding. Neuroimaging and neuropsychological studies of semantically congruent cross-modal processing has led to a Conceptual Structure Account model (Tyler and Moss 2001; Taylor et al. 2009), suggesting that objects in different categories can be characterized by the number and statistical properties of their constituent features (i.e., its depth), and this model points to the anterior temporal poles as “master binders” of such audio-visual information.

Correlating static-visual images with sound could be argued to require a more cognitive learning process than perceptually observing dynamic-visual events as they unfold and provide more intermodal-invariant information correlated with ongoing acoustic information. Thus, it was somewhat surprisingly that the static-visual stimuli preferentially recruited of the bilateral planum temporale (Fig. 3*E*, pink hues), in locations close to secondary auditory cortices, rather than in the temporal poles. However, this may relate to depth-of-encoding issues. The audio-visual stimuli used in many of the included studies used a relatively basic level of semantic matching (stimuli and tasks), which may have masked more subtle or widespread activation in inferotemporal cortices (e.g., temporal poles).

One possibility is that the pSTS complexes may represent intermediate processing stages that convey dynamically matched audio-visual congruent interaction information to the temporal poles, while the bilateral planum temporale regions may represent parallel intermediate processing stages that convey semantically congruent audio-visual information derived from learned associations of sound with static (iconic) images referring to their matching source. Overall, this interpretation supports the tenet from unisensory systems “that parallel hierarchical pathways process increasing information content,” but here including two parallel multisensory processing pathways mediating the perception of audio-visual interaction information as events that are physically matched from a bottom-up perspective versus learned to be semantically congruent.

#### Semantic Processing and Neurocomputational Models of Cognition

Several mechanistic models regarding how and why semantic knowledge formation might develop in the brain includes the concept of hubs (and connector hubs) in brain networks (Damasio 1999; Sporns et al. 2007; Pulvermuller 2018), which are thought to allow for generalizations and the formation of categories. As such, the roughly six basic ROIs emerging from the present meta-analysis study (left and right pSTS complexes, left and right planum temporale, left fusiform, and right anterior insula) were of particular interest.

With regard to double-dissociations of cortical function, the right anterior insula and left fusiform ROIs had relatively small volumes, and thus may be considered less robust by some meta-analysis standards (also see Limitations). Nonetheless, these preliminary findings provide at least moderate support for a taxonomic neurobiological model for processing different categories of real-world audio-visual events (Fig. 1), which is readily amenable to testing with neurocomputational models and future hypothesis-driven multisensory processing studies. For instance, one might directly assess whether the different ROIs have functionally distinct characteristics as connector hubs for semantic processing with activity dynamics that are functionally linking action perception circuits (APCs) at a category level (Pulvermuller 2018). Additionally, one may test for functional connectivity pattern differences across these ROIs (e.g., resting state functional connectivity MRI) in neurotypical individuals relative to various clinical populations. Overall, the results indicating that different semantic categories of audio-visual interaction events may be differentially processed along different brain regions supports the tenet that “categorical perception emerges in neurotypical listeners [observers],” but here applying to the realm of cortical representations mediating multisensory object information. It remains unclear, however, whether this interpretation regarding categorical perception would provide greater support for domain-specific theoretical models, as proposed for some vision-dominated categories, such as the processing of faces, tools, fruits and vegetables, animals, and body parts (Damasio et al. 1996; Caramazza and Shelton 1998; Pascual-Leone and Hamilton 2001; Mahon and Caramazza 2005; Mahon et al. 2009) or for sensory-motor property-based models that develop because of experience (Lissauer 1890/1988; Barsalou et al. 2003; Martin 2007; Barsalou 2008), or perhaps some combination of both.

##### Limitations

While this meta-analysis study revealed significant dissociations of cortical regions involved in different aspects of audio-visual interaction processing, at a more detailed or refined level there were several limitations to consider. As with most meta-analyses, the reported results were confined only to published “positive” results, and tended to be biased in examining topics (in this case sensory stimulus categories) that typically have greater rationale for being studied (and funded). In particular, the categories of living things (humans) and/or vocalizations (speech) are simply more thoroughly studied as socially- and health-relevant topics relative to the categories of nonliving and nonvocal audio-visual stimuli, as evident in the numbers of studies listed in the provided tables. Because there were fewer numbers of studies in some semantic categories, double-dissociation differences could only be observed in some contrast meta-analyses when using uncorrected *P*-values, a statistical correction process that to date remains somewhat contentious in the field of meta-analyses. The biases in stimuli commonly used also led to the limitation that there would be greater heterogeneity of, for instance, nonliving audio-visual sources and action events devoid of any vocalizations. This precluded examination of subcategories such as environmental sources, mechanical (human-made) audio-visual sources, versus “artificial” events (being computer-derived or involving illusory sources), which limited a more thorough testing of the taxonomic model (Fig. 1) being investigated.

At a more technical level, other potential limitations included methodological differences across study designs, such as 1) differences in alignment methods, 2) imaging large swaths of brain rather than truly “whole brain” imaging, and 3) potential inclusion of participants in more than one published study (which was not accessible information). Together, these limitations may constitute violations of assumptions by the ALE meta-analysis processes. Nonetheless, the modest support for our first two hypotheses and strong support for our third hypothesis should merit future study to validate and/or refine these basic cortical organization tenets and neurobiological taxonomic model.

## Conclusion

This study summarized evidence derived from meta-analyses across 137 experimental paradigms to test for brain organizations for representing putative taxonomic boundaries related to perception of audio-visual events at a category-level. The semantic categories tested were derived from an ethologically and evolutionarily inspired taxonomic neurobiological model of real-world auditory event perception. The outcomes provided novel, though tentative support for the existence of double-dissociations mediating processing and perception around semantic categories, including 1) living versus nonliving (artificial) audio-visual events, and 2) vocalization versus action audio-visual events. The outcomes further provided strong support for a double-dissociation for processing 3) dynamic-visual (mostly natural events) versus static-visual (including artificial) audio-visual interactions. Together, these findings were suggestive of parallel hierarchical pathways for processing and representing different semantic categories of multisensory event types, with embodiment strategies as potential underlying neuronal mechanisms. Overall, the present findings highlighted where and how auditory and visual perceptual representations interact in the brain, including the identification of a handful of cortical hubs in Figure 3*C*–*E* that are amenable to future neurocomputational modeling and testing of semantic knowledge representation mechanisms. Exploration of these and other potential multisensory hubs will be important for future studies addressing why specific brain regions may typically develop to process different aspects of audio-visual information, and thereby establish and maintain the “multisensory brain,” which ultimately subserves many of the complexities of human communication and social behavior.

## Supplementary Material

AppendixA_201226_tgab002

AVmeta2020_Fig3A_1a_Combine_CongrIncong_Uncorr_p999-Copy_tgab002

AVmeta2020_Fig3B_2a_CongruentOnly_FWE05_10k_tgab002

AVmeta2020_Fig3B_2b_IncongruentOnly_FWE05_10k_tgab002

AVmeta2020_Fig3B_2c_ContrastCongruentVsIncongruent_10k_Puncorr_Z_p05_tgab002

AVmeta2020_Fig3B_2d_Contrast_IncongruentVsCongruent_10k_Puncorr_Z_p05_tgab002

AVmeta2020_Fig3C_3a_LivingOnly_FWE05_10k_tgab002

AVmeta2020_Fig3C_3b_NonLivingOnly_FWE05_10k_tgab002

AVmeta2020_Fig3C_3c_Contrast_LivingVsNonLiving_10k_uncorr_Z_p05_tgab002

AVmeta2020_Fig3C_3d_Contrast_NonLivingVsLiving_10k_uncorr_Z_p05_tgab002

AVmeta2020_Fig3D_4a_Vocalizations_FWE05_10k_tgab002

AVmeta2020_Fig3D_4b_ActionEvents_FWE05_10k_tgab002

AVmeta2020_Fig3D_4c_Contrast_VocalizationsVsActions_10k_uncorr_Z_p05_tgab002

AVmeta2020_Fig3D_4d_Contrast_ActionsVsVocalizations_10k_uncorr_Z_p05_tgab002

AVmeta2020_Fig3D_4e_EmotionalLiving_FWE05_10k_tgab002

AVmeta2020_Fig3E_9a_DynamicVisual_FWE05_10k_tgab002

AVmeta2020_Fig3E_9b_StaticVisual_FWE05_10k_tgab002

AVmeta2020_Fig3E_9c_Contrast_DynamicVsStaticVisual_10k_uncorr_Z_p05_tgab002

AVmeta2020_Fig3E_9d_Contrast_StaticVsDynamicVisual_10k_uncorr_Z_p05_tgab002

LRV_MPRAGE+tlrc_BRIK_tgab002

LRV_MPRAGE+tlrc_tgab002

## Data Availability

The data that support the findings of this study are available from the corresponding author upon reasonable request.

## Appendix A

List of all 137 experimental coordinates from the 82 studies after converting all to afni-TLRC coordinates using GingerALE software. The number of subjects are also indicated. The coordinate sets were used to derive all of the meta analyses of the present study.
